# Identification and characterization of highly versatile peptide-vectors that bind non-competitively to the low-density lipoprotein receptor for *in vivo* targeting and delivery of small molecules and protein cargos

**DOI:** 10.1371/journal.pone.0191052

**Published:** 2018-02-27

**Authors:** Marion David, Pascaline Lécorché, Maxime Masse, Aude Faucon, Karima Abouzid, Nicolas Gaudin, Karine Varini, Fanny Gassiot, Géraldine Ferracci, Guillaume Jacquot, Patrick Vlieghe, Michel Khrestchatisky

**Affiliations:** 1 VECT-HORUS SAS, Marseille, France; 2 Aix Marseille Univ, CNRS, NICN, Marseille, France; 3 Aix Marseille Univ, CNRS, CRN2M, Marseille, France; 4 Aix Marseille Univ, CNRS, INP, Inst Neurophysiopathol, Marseille, France; Helsingin Yliopisto, FINLAND

## Abstract

Insufficient membrane penetration of drugs, in particular biotherapeutics and/or low target specificity remain a major drawback in their efficacy. We propose here the rational characterization and optimization of peptides to be developed as vectors that target cells expressing specific receptors involved in endocytosis or transcytosis. Among receptors involved in receptor-mediated transport is the LDL receptor. Screening complex phage-displayed peptide libraries on the human LDLR (hLDLR) stably expressed in cell lines led to the characterization of a family of cyclic and linear peptides that specifically bind the hLDLR. The VH411 lead cyclic peptide allowed endocytosis of payloads such as the S-Tag peptide or antibodies into cells expressing the hLDLR. Size reduction and chemical optimization of this lead peptide-vector led to improved receptor affinity. The optimized peptide-vectors were successfully conjugated to cargos of different nature and size including small organic molecules, siRNAs, peptides or a protein moiety such as an Fc fragment. We show that in all cases, the peptide-vectors retain their binding affinity to the hLDLR and potential for endocytosis. Following i.v. administration in wild type or *ldlr-/-* mice, an Fc fragment chemically conjugated or fused in C-terminal to peptide-vectors showed significant biodistribution in LDLR-enriched organs. We have thus developed highly versatile peptide-vectors endowed with good affinity for the LDLR as a target receptor. These peptide-vectors have the potential to be further developed for efficient transport of therapeutic or imaging agents into cells -including pathological cells—or organs that express the LDLR.

## Introduction

Targeted organ delivery and expedient biotransport of drugs are challenging goals for the pharmaceutical industry and the search for alternative modes of drug delivery has developed as an active field of research. A promising strategy is the development of uptake-facilitating ligands that target specific receptors involved in endocytosis [1, 2].

The low-density lipoprotein receptor (LDLR) family is composed of a class of single transmembrane glycoproteins recognized as cell surface endocytic receptors that bind apolipoprotein complexes, that may elicit signal transduction upon binding of extracellular ligands, and that internalize these ligands for intracellular processing and/or degradation by lysosomes [3]. Structurally, members of the LDLR family share homology within their extracellular domains, which are highlighted by the presence of clusters of ligand-binding repeats. Among these receptors, the LDLR binds cholesterol-carrying lipoprotein particles such as LDL [4]. Cholesterol plays several structural and metabolic roles that are vital. It is found in the plasma membrane of cells, concentrates in rafts and caveolae which are sphingolipid-rich domains, and modulates membrane fluidity [5]. LDL are internalized by endocytosis followed by conversion to an early endosome, where the low-pH environment results in LDL release, and lysosomal degradation, while the receptor is recycled to the cell surface [6]. Alternatively, upon binding to the PCSK9 protein, the LDLR-PCSK9 complex is directed to lysosomes for degradation, thereby leading to LDLR down-regulation [7].

The LDLR is expressed in the parenchyma of different organs [8, 9], for instance in the liver where a large part of body cholesterol is synthesized. Cholesterol is also an obligatory precursor for steroid hormone production in steroidogenic tissues, such as gonads, brain, placenta, and the adrenal glands. The LDLR is also present throughout the intestine, the jejunum, the duodenum, and the colon [10]. Osteoclast formation and viability depends on cholesterol and lipoprotein delivery via the LDLR [11]. In the eye, the retina is also capable of rapid uptake of circulating LDL via an LDLR-mediated process [12]. There is also evidence that LDLR expression is increased in cancer cells (reviewed in [13, 14]), presumably due to the high need of cancer cells for cholesterol. Finally, the LDLR is expressed in endothelial cells and like several other receptors of the blood-brain barrier (BBB) such as the transferrin receptor (TfR), insulin receptor (IR), low density lipoprotein receptor-related protein 1 (LRP1) [2, 15–17] has been described to undergo receptor-mediated transcytosis (RMT) [18, 19], and to transport payloads into the brain [17, 20, 21].

Using in vitro selection of large peptide libraries and medicinal chemistry-based rational design and optimization (overall approach is schematized in S1 Fig), we describe here the identification of a family of peptides with the following requirements: i) unambiguous targeting of the extracellular domain of the human LDLR (hLDLR), ii) conserved affinity for rodent receptors to allow preclinical studies, iii) minimal sized peptide-vectors, preferentially cyclic and chemically optimized for increased binding affinity, iv) absence of competition with the binding of LDL, the main endogenous ligand, and v) in vitro and in vivo validation of the peptide-vectors on a ldlr-/- background.

We identified peptides that meet the above-mentioned requirements. We showed that they are highly versatile and can be conjugated to a large variety of molecules, ranging from small organic molecules to siRNAs, peptides and proteins, while retaining their potential to bind the LDLR and to be internalized by cells. When peptide-vectors alone or conjugated to a cargo were administered in vivo in mice, we showed significant accumulation in LDLR-enriched tissues when compared to control molecules or administration in ldlr-/- mice.

## Materials and methods

### Animals

Procedures involving animals conform to National and European regulations (EU Directive N°2010/63) and to authorizations delivered to our animal facility (N° C13 055 08) and to the project (N° 00757.02) by the French Ministry of Research and Local Ethics Committee. All efforts were made to minimize animal suffering and reduce the number of animals used. We used C57Bl/6 WT male mice (from Elevage Janvier, St. Berthevin, France).

### Cloning and production of recombinant proteins

#### Cloning of the human, mouse and rat LDLR and of the human transferrin receptor in fusion with EGFP

Full length coding regions for hLDLR, mLDLR, rLDLR and hTfR were amplified by RT-PCR from human (Clontech laboratories, Mountain View, CA, USA), mouse and rat total brain RNA using the following pairs of forward (F) and reverse (R) primers for hLDLR, mLDLR, rLDLR and hTfR (Table 1).

**Table 1:** Sequences of RT-PCR primers.

hLDLR	(F)ATATATAAGCTTCGAGGACACAGCAGGTCGTGAT (R)TTAATTGTCGACCACGCCACGTCATCCTCCAGACT
mLDLR	(F)ATATATAAGCTTGACGCAACGCAGAAGCTAAG (R)TTAATTGGTACCGTTGCCACATCGTCCTCCAG
rLDLR	(F) ATATATAAGCTTACATTTCGGGTCTGTGATCC (R) TTAATTACCGGTCATGCCACATCATCCTCCAG
hTfR	(F) ATATATGAATTCGGCTCGGGACGGAGGACGC (R) TTAATTGTCGACAGAACTCATTGTCCCAACCGTCAC

The PCR products were cloned in frame with EGFP in the pEGFP-N1 or C1 vectors (Clontech). Plasmid constructs were named hLDLR-pEGFP, mLDLR-pEGFP, rLDLR-pEGFP and hTfR-pEGFP. All constructs were fully sequenced on both strands.

#### Subcloning of functional domains of the hLDLR extracellular domain

PCR products were generated that encode the intracellular and transmembrane domains (residues 786 to 860) of the hLDLR, encompassing either: i) a HA tag (YPYDVPDYA) and signal peptide; ii) the O-glycosylation domain alone (residues 728 to 785), HA tag and signal peptide; iii) the O-glycosylation domain, the EGF-precursor homology domain (residues 313 to 785), HA tag and signal peptide; iv) the O-glycosylation domain, the EGF-precursor homology domain, the ligand binding domain (residues 22 to 785), HA tag and signal peptide. These PCR products were cloned into the pEGFP-N1 plasmid to express these constructs in phase with EGFP. All clones were fully sequenced on both strands. Constructs were named respectively hLDLR-ΔECD, hLDLR-ΔEGF, hLDLR-ΔLBD, hLDLR-FL. The HA tag was used to verify appropriate extracellular distribution of the extracellular domains following cell transfection.

#### Cloning of the hLDLR extracellular domain and VH434 in phase with a human antibody IgG1-Fc fragment

DNA fragments encoding the extracellular domain of hLDLR (residues 1 to 788) and the VH434 peptide (CMPRLRGC) we identified were amplified by PCR and cloned into the pINFUSE-IgG1-Fc2 vector (InvivoGen, Toulouse, France) in order to encode a hLDLR extracellular domain encompassing in its C-ter a human IgG1-Fc Fragment on the one hand, and a human IgG1-Fc Fragment encompassing in its C-ter the VH434 peptide on the other. The plasmid constructs were fully sequenced and named phLDLR-Fc and pVH434-Fc. The pINFUSE-IgG1-Fc2 control plasmid was named pFc.

#### Production of fusion proteins

2 x 106 HEK 293 cells (ATCC number: CRL-1573) were transfected with 24 μg of pFc or phLDLR-Fc or pVH434-Fc using lipofectamine 2000 transfection reagent in 10 ml of OptiMEM medium. Media was changed after 6 hrs of transfection and replaced by DMEM glutaMax supplemented with Insulin Transferrin Selenium 1X (all reagents from Life Technologies, Saint-Aubin, France) and a cocktail of protease inhibitors (1/400, Sigma-Aldrich, Saint-Quentin Fallavier, France). After 72 hrs, supernatants were recovered and concentrated to 1 ml with Ultra Cell Amicon columns with a cut-off of 10 kDa (Millipore, Billerica, MA, USA) or were purified using the Montage antibody protein A PROSEP A kit (Millipore) according to the manufacturer’s recommendations. The purified Fc and Fc-fusion proteins were quantified by an anti-Fc ELISA test developed in house and were checked for their ability to bind CHO-hLDLR-EGFP overexpressing cells.

### Pull down assay

Epoxy-270 Dynabeads (4 x108 M) (Life Technologies) were washed with 0,1 M sodium phosphate buffer pH 7.4 to obtain a bead concentration of 1.2x109 /mL. Beads were coated overnight at 37°C with peptides we identified (VH445-S-Tag or the scrambled VH4Sc-S-Tag) diluted in 0,1M sodium phosphate buffer pH 7.4 in the presence of 1M ammonium sulfate. The supernatant was removed and coated beads were washed with PBS + BSA 0,5%. Concentrated supernatants of HEK cells containing hLDLR-Fc or Fc were added to the coated beads at a concentration of 1.2x109 beads/mL and incubated for 1 hr at 4°C. Beads were next washed with PBS and eluted with 100 μL of Laemmeli buffer. The eluted and washed fractions were collected and analyzed by western blot.

### Establishment of stable cell lines

One day before transfection, Chinese Hamster Ovary cells (CHO-K1, ATCC number CCL-61TM) were seeded and cells were transfected using JetPei (Polyplus Transfection, Strasbourg, France) with the different plasmid constructs according to the manufacturer’s instructions. Forty-eight hrs after transfection, 800 μg/mL geneticin (Life Technologies) was added to the growth medium. One week after the beginning of the selection, individual fluorescent cells were seeded in 96 well plates. Isolated EGFP expressing clones were selected, amplified and tested for the expression of the fusion proteins of interest by western blotting and immunocytochemistry.

### Preparation of cell membrane extracts and tissue lysates

In order to assess expression of the different constructs following transient transfection or in stable cell lines we generated, cells were washed with phosphate-buffered saline (PBS, Life technologies), scrapped, centrifuged and treated as recommended in the ProteoExtract Subcellular Proteome Extraction Kit (Calbiochem, La Jolla, CA, USA) to prepare total cell membranes. In order to detect the Fc conjugates in tissues of injected mice, organs (liver and adrenals), were mechanically dissociated in PBS 0.1% Triton X100, snap frozen, thawed and sonicated 10 x 10 s. Protein content in membrane and tissue extracts were quantified using the BioRad DC Protein Assay (Bio-Rad, Hercules, CA, USA) following manufacturer’s instructions.

### Western Blot assays

One μg of cell membrane extracts, 30μl of pull down eluted fraction, 35 μg of adrenal and 100 μg of liver were separated by sodium dodecyl sulfate polyacrylamide gel electrophoresis (SDS-PAGE) on 8% polyacrylamide gels, and transferred onto nitrocellulose membranes (Amersham Biosciences, Buckinghamshire, UK). Proteins were probed with antibodies against EGFP (mouse 1/1000, Roche, Basel, Switzerland), hLDLR (1/800, rabbit, Abcam, Cambridge, UK), mLDLR (1/1000, goat, R&D systems, Minneapolis, MN) and hFc (1/1000, mouse, Jackson ImmunoResearch, Baltimore, MD, USA). After incubation with primary antibodies, membranes were incubated with a peroxidase conjugated secondary antibody (donkey, 1/2000, Jackson ImmunoResearch). Finally, proteins were detected using a chemiluminescence kit (Roche Diagnostics) and revealed with the G:Box chemi XX6 system (Syngene, Cambridge, UK). The signal intensity of bands was quantified using Image J software when necessary.

### Biopanning with phage displayed peptide libraries and biopanning competition assays

Different phage display libraries (7- and 12-mer linear, 7-mer Cys, all from New England Biolabs, Ipswich, UK) as well as 15-mer and 6-mer linear libraries from the Smith Laboratory (Division of Biological Science University of Missouri, Columbia, MS, USA) were used for biopanning. Following subtraction of the libraries (2.1011 phage) on naive CHO-EGFP cells for 1 h, biopanning was performed on the CHO-hLDLR-EGFP cell line. Cells were pelleted, phage were eluted in elution buffer (0.2M glycine/HCl, pH 2.2) and were amplified in Luria Bertani medium containing E. Coli ER2738 or K91BK. Phage were precipitated and, following resuspension, were used for additional rounds of biopanning. After each round of biopanning, the final eluates were tittered, amplified in E. coli, and plated. Following 3 to 5 rounds of biopanning, individual plaques were subjected to PCR amplification with primers that span the region encoding the peptides presented by the phage. PCR products were sequenced. For the biopanning competition assays 2.1010 phage of each of the clones presenting peptides were mixed and added to confluent CHO-hLDLR-EGFP cells. Cells were washed and eluted as described above. Eluted phage were sequenced to identify phage clones that out-competed the others.

### Flow cytometry

Phage (1.10^10^) presenting peptides that bind the LDLR were incubated on ice for 20 min with 1.10^6^ HUVEC cells (Lonza) in the absence or presence of LDL at 0.5 mg/mL. After several washes, cells were incubated with an anti-M13 bacteriophage (mouse, 1/1000, Abcam) and an anti-hLDLR (rabbit, 1/50, Abcam), followed by incubation with A488-conjugated (mouse, 1/800, Life technologies) and A647-conjugated (rabbit, 1/800, Life technologies) antibodies. A FACS analysis was performed on a FacsCanto (Becton Dickinson, Franklin Lakes, NJ, USA) using the BD FACSDiva Software. The number of positive cells in each assay was standardized to 5000. Results were expressed in arbitrary units corresponding to the mean of fluorescence.

### Immunocytochemistry

Cells alone or incubated 1 hr at 37°C with phage or peptide conjugates (10μM for S-Tag, fluorescent or siRNA conjugates and 10nM for Fc conjugates) were washed and subsequently fixed with 4% paraformaldehyde, washed again and incubated for 30 min at room temperature in PBS containing 3% BSA with or without 0.1% triton X-100 (Sigma-Aldrich). Cells were washed with PBS and incubated for 1 hr with primary antibodies directed against the hLDLR (rabbit, 1/100, Acris, Herford, Germany), against M13 bacteriophage (mouse, 1/3000, Sigma-Aldrich), against the S-Tag (goat, 1/250, Abcam) or against the Fc (mouse, 1/100, Jackson ImmunoResearch), followed by 3 washes with PBS and incubation with Alexa fluor 488, 594 or 647 anti-rabbit, anti-mouse or anti-goat secondary antibodies (1/800, Invitrogen). Cell nuclei were labeled with Hoechst #33258 (0.5 μg/mL, Invitrogen). In the experiments performed to validate functionality of the hLDLR receptors expressed by the CHO-hLDLR-EGFP cell line, binding of DiI-LDL (15 μg/mL) was assessed on live cells. In some experiments, binding and endocytosis of macromolecular complexes were assessed on live CHO-hLDLR-EGFP cells. These complexes result from the co-incubation and interaction for 1 hr at 37°C of 10 μM VH411-S-Tag peptide or VH4127-S-Tag, ApoB-S-Tag, ApoE1-S-Tag, ApoE2-S-Tag and VH411Sc-S-Tag peptides with the anti-S-Tag antibody (1/200) and the Alexafluor 647 or 594-conjugated secondary antibodies (1/800). For co-incubation with the VH411-S-Tag peptide, cells were concomitantly exposed to 10 μg/mL of DiI-LDL. Following fixation, cell nuclei were labeled with Hoechst#33258. Mounted slides were acquired with a Leica TCS SP2 confocal microscope (Leica Microsystems, Heidelberg, Germany). Images were analyzed using ImageJ software and quantification of co-localization between two markers was performed using the JaCoP plug-in, by calculating Mander’s correlation coefficients from unmodified images.

### Chemistry of peptides and peptide conjugates: Synthesis and characterization

ApoB-S-Tag, ApoE1-S-Tag and ApoE2-S-Tag (respectively Pr-SVIDALQYKLEGTTRLTRKRGLKLATALSLSNKFVEGS-GGG-KETAAAKFERQHMDS-NH2, Spencer and Verma, 2007; Pr-TEELRVRLASHLRKLRKRLLRDA-GGG-KETAAAKFERQHMDS-NH2; Pr-LRKLRKRLLLRKLRKRLL-GGG-KETAAAKFERQHMDS-NH2, Sarkar et al., 2011), were synthesized by Proteogenix (Oberhaus-bergen, France). All the other peptides and peptide-conjugates were synthesized in house as described below. All the peptides and peptide-conjugates were obtained with a purity >95% which was assessed by High Performance Liquid Chromatography (HPLC) on a C18 column (Kinetex C18, 5μm, Phenomenex). Identity of the molecules was assessed either by MALDI-TOF or ESI mass spectrometry (mass analysis results are given in S1 Table).

#### Solid Phase Peptide Synthesis (SPPS)

The peptide-vectors were synthesized using Fmoc SPPS methodology with an automated microwave peptide synthesizer (CEM Corporation, Matthews, NC, USA) as we previously described (Malcor et al, 2012). After acid cleavage of the peptidyl resin, cyclization was performed on crude peptides by intramolecular disulfide bridge formation using K3[Fe(CN)6] as a mild oxidating reagent between the first Cys or D-Cys residue and the last Cys or Pen residue. Cyclic peptides were then purified and their homogeneity and identity were successfully assessed by analytical RP-HPLC and MALDI-TOF mass spectrometry. Depending on the final use of the peptides, their N-ter was either propionylated (VH411, VH434, VH445, VH4127, VH411-S-Tag, VH445-S-Tag, VH4127-S-Tag, siGLO CylcophilineB-VH4127, Fc-(VH4Sc)2, Fc-(VH4127)2, VH445 dimer and VH4127 dimer) or kept free for further conjugation (Cy5.5-PEG6-VH4127) while the C-ter was either carboxamide (VH411, VH434, VH445, VH4127, VH411-S-Tag, VH445-S-Tag, VH4127-S-Tag, Cy5.5-PEG6-VH4127) or carboxylic acid for further functionalization (siGLO CyclophilineB-VH4127, Fc-(VH4Sc)2, Fc-(VH4127)2, VH445 and VH4127 dimers).

#### Cy5.5 conjugation: Synthesis of Cy5.5-PEG6-VH4127

For Cy5.5-PEG6-VH4127 preparation, cyclic H-PEG6-VH4127-NH2 was first synthesized. NHS ester chemistry was used to fluorescently label the N-terminus of this peptide with Cy5.5 (6S-IDCC NHS ester, Mivenion, Berlin, Germany). After purification, homogeneity and identity of this peptide were successfully assessed by analytical RP-HPLC and MALDI-TOF mass spectrometry.

#### Fc conjugation: Synthesis of Fc-(VH4Sc)_2_, Fc-(VH4127)_2_

Fc conjugation was performed using the heterobifunctional linker sulfo-SMCC (Thermo Fischer Scientific) as a spacer between thiol-modified peptides and the IgG1-Fc fragment. This crosslinking reagent contains two reactive moieties: a NHS group that reacts with Fc fragment lysine side chains, and a maleimide group able to react with sulfhydryl-peptides. In this aim Pr-VH4Sc-G-OH and Pr-VH4127-G-OH were synthesized, cyclized, purified and further functionalized on their C-ter with cysteamine via: i) activation of the first Gly residue with PyBop/DIEA in DMF and reaction with 2-tritylthio-1-ethylamine hydrochloride (Trt-cysteamine) and ii) removal of cysteamine trityl protections in acidic conditions (DCM/TIS/TFA: 3/1/1). A Gly residue was inserted at the C-ter position to enable functionalization while avoiding racemization. After RP-HPLC purification peptide purity and identity were successfully assessed by analytical HPLC and MALDI-TOF mass spectrometry. Conjugation of the sulfhydryl Pr-VH4Sc-G-C(O)NH-CH2-CH2-SH and Pr-VH4127-G-C(O)NH-CH2-CH2-SH peptides to human IgG1-Fc fragment was performed in two steps. In a first step the IgG1-Fc fragment was allowed to react with sulfo-SMCC (12 eq.) to obtain activated proteins against sulfhydryls. This was possible considering that the IgG1-Fc fragment contains no free cysteine. Excess of sulfo-SMCC and its by-products were removed using a desalting column (Pierce Dextran desalting column). In a second step the resulting maleimide-functionnalized Fc and the sulfhydryl peptides were mixed together using a 5 molar excess of peptide. Excess of peptide was removed by purification on a desalting column (Pierce Dextran desalting column). Conjugate concentrations were determined using the anti-Fc ELISA assay we developed. The number of peptides per dimerized Fc was quantified by MALDI-TOF mass spectrometry. The conjugates were checked for binding to the human, mouse and rat LDLR-expressing CHO cells.

#### siGLO Cyclophiline B conjugation: Synthesis of siGLO Cyclophiline B-VH4127 and siGLO Cyclophiline B-VH4Sc

Conjugation to siRNA was performed in 2 steps with a protocol similar to the one set up for Fc conjugation using the heterobifunctional linker sulfo-SMCC and the same sulfhydryl Pr-VH4Sc-G-CH2-NH2-NH2-SH and Pr-VH4127-G-CH2-NH2-NH2-SH peptides. The Cyclophilin B targeting siRNA bearing a DY547 modification on the 5’ moiety of the sense strand and a N6 (amino, 6 carbon) linker on the 3’ moiety of the antisense strand (GE Dharmacon, Lafayette, USA) was dissolved at 2 mM in water. The siRNA was then diluted at 2 mg/mL in phosphate buffer pH 8.2 and incubated for 90 min at room temperature with 100 eq. of sulfo-SMCC followed by a 15 min incubation at 80°C. The reaction mixture was slowly cooled to room temperature to allow RNA duplex formation. Functionalized siRNA was precipitated by the addition of 0.3 vol. NaCl 1M and 2.5 vol. of absolute ethanol and collected by centrifugation. The pellet was then washed with 70% EtOH, dried, dissolved in PBS pH 7.2 at a 2 mg/mL concentration and incubated for 2 hrs at room temperature with 5 eq. of each thiol-modified peptide. The siGLO Cyclophiline B-VH4127 and siGLO Cyclophiline B-VH4Sc conjugates were precipitated as above. Conjugates were purified by RP-HPLC and analyzed using SDS-page and RP-HPLC analysis.

#### Synthesis of VH445 and VH4127 dimers: Synthesis of (Pr-VH445-G-CH_2_CH_2_)2-N-CH_2_CH_2_-NH_2_ and (Pr-VH4127-G-CH_2_CH_2_)2-N-CH_2_CH2-NH_2_

Pr-VH445-G-OH or Pr-VH4127-G-OH were synthesized, cyclized, purified, pre-activated with PyBop/DIEA in DMF and then added to a Tris(2-aminoethyl)amine solution in dry DMF at 0°C. The reaction mixtures were then allowed to stir overnight at room temperature. Reaction completion was monitored by analytical RP-HPLC chromatography. Pure dimers were obtained as white powders after RP-HPLC purification. Dimer purity and identity were successfully assessed by analytical HPLC and MALDI-TOF mass spectrometry.

### Surface Plasmon Resonance analysis

Recombinant human and mouse LDLR (His-tagged) were purchased from Sino Biological (Beijing, China). Interaction of ligands with LDLR was tested at 25°C using a Biacore T200 (GE Healthcare) and 50 mM HEPES-NaOH pH7.4, 150 mM NaCl, 0.005% Tween-20, 50μM EDTA as running buffer. hLDLR or mLDLR were immobilized on a NiHC sensor chip (Xantec, Dusseldorf, Germany) at a density of 35–60 fmol/mm^2^. Binding to LDLR-coated flow cells was corrected for non-specific binding to uncoated flow cells. The single-cycle kinetic method was used to measure the affinity of ligands with LDLR. Ligands were diluted in running buffer and injected sequentially 2 minutes at 30 μl/min using increasing concentrations. Blank run injections of running buffer were performed in the same conditions before ligand injection. Double-subtracted sensorgrams were globally fitted with the 1:1 Langmuir binding model from Biacore T200 Evaluation version 2.0 except for the VH445 and VH4127 dimers for which the bivalent analyte model was applied. Data are representative of at least three independent experiments.

### ELISA Assays

The anti-S-Tag ELISA assay was used to assess the S-Tagged peptides binding to CHO-hLDLR-EGFP cells (800 000 cells per well in 6 well plates) at a concentration of 10 μM during 1 hr at 37°C, 5% CO_2_. The anti-Fc ELISA assay was used to assess Fc conjugate binding to CHO-hLDLR-EGFP cells (10 nM) or distribution in mouse tissue 15 min and 2 hrs post-injection at a dose of 400 pmole/mouse.

The day before the assay, microtitration plates were coated with an anti-S-Tag capture antibody (goat, 2μg/mL, Abcam) or anti-Fc antibody (mouse, 10μg/mL, Jackson ImmunoResearch) in NaHCO3 (0.1M) buffer and incubated overnight. The day of the assay, the plate was blocked for 1 hr at 4°C with NaHCO_3_ -BSA 0.5% buffer. After six washes with PBS-Tween 0.05% (PBST), the samples (i.e CHO-hLDLR-EGFP cell lysates obtained after incubation of cells with VH4127-S-Tag or VH4Sc-S-Tag peptides, or the tissue lysates of (VH4127)_2_-Fc injected mice) were incubated for 1 hr and overnight respectively. After six washes with PBST, the anti-S-Tag detection antibody (rabbit, 0.2μg/mL, Abcam) was incubated for 1 hr. Unfixed antibody was removed by six new PBST washes and a HRP conjugated anti- antibody (rabbit, 1/2000, Jackson ImmunoResearch) was incubated for an additional hour. Revelation of the plate was performed using OPD substrate (Sigma Aldrich) after six PBST washes and OD reading at 492nM.

### In vivo distribution analysis of Fc conjugates

Wild type C57Bl/6J (from Elevage Janvier) and *ldlr*-/- (B6.129S7-Ldlrtm1Her/J, The Jackson Laboratory, Bar Harbor, ME, USA) male mice aged 8 to 10 weeks were used for all studies. Mice were injected in the tail vein with 100 μL of Fc conjugates at 0.5 mg/kg. After 15 min or 2 hrs, mice were deeply anesthetized with a cocktail of ketamine (100 mg/kg)/xylasine (10 mg/kg) (Sigma Aldrich). Whole blood was collected in heparinized tubes (Sigma Aldrich) and plasma was isolated after 15 min centrifugation at 5000 g. Mice were transcardially perfused with 50 mL of NaCl 0.9%. Organs were extracted, weighed, and homogenized in PBS/0.1% Triton (Sigma Aldrich) in PBS containing a protease inhibitor cocktail (Sigma Aldrich). Organ homogenates were frozen at -80°C for 12 hrs before sonication 3 x 10 s and clarification of the tissue lysates was performed by centrifugation for 15 min at 20000 g. Fc concentrations in the isolated supernatants were measured with an anti-Fc ELISA as described above.

### Immunohistochemistry of Fc conjugates

For immunohistochemistry, transcardiac NaCl 0.9% perfusion was followed by 50 mL of PBS 1X- 4% paraformaldehyde (PFA). Mice organs were then removed and snap frozen in cold isopentane. Cryostat (Leica CM-3050-S) sections (14 μm thick) were stored at −80°C. Organ sections were first permeabilized and blocked for 1 hr at room temperature using a solution of PBS 1X, 0.1% Triton X-100 and 3% Bovine Serum Albumin (BSA). Sections were then incubated overnight at 4°C with anti-Fc-A594 (goat, 1/200, Jackson ImmunoResearch) and anti-mLDLR (goat 1/200, R&D Systems), followed by anti-goat-Alexa 488 (donkey, 1/800, Life Technologies) for 1 hr at room temperature. Nuclei were stained with Hoechst (0.5 μg/mL, Life Technologies). Omission of the primary antibody was used as control and no immunostaining was observed. Sections were mounted using Prolong Gold Antifading reagent (Life Technologies) on Superfrost glass slides. Images were taken and processed using a confocal microscope (LSM 700) and Zen software (Zeiss, Jena, Germany).

## Results

### Identification of peptides that bind the hLDLR

**Fig 1:**
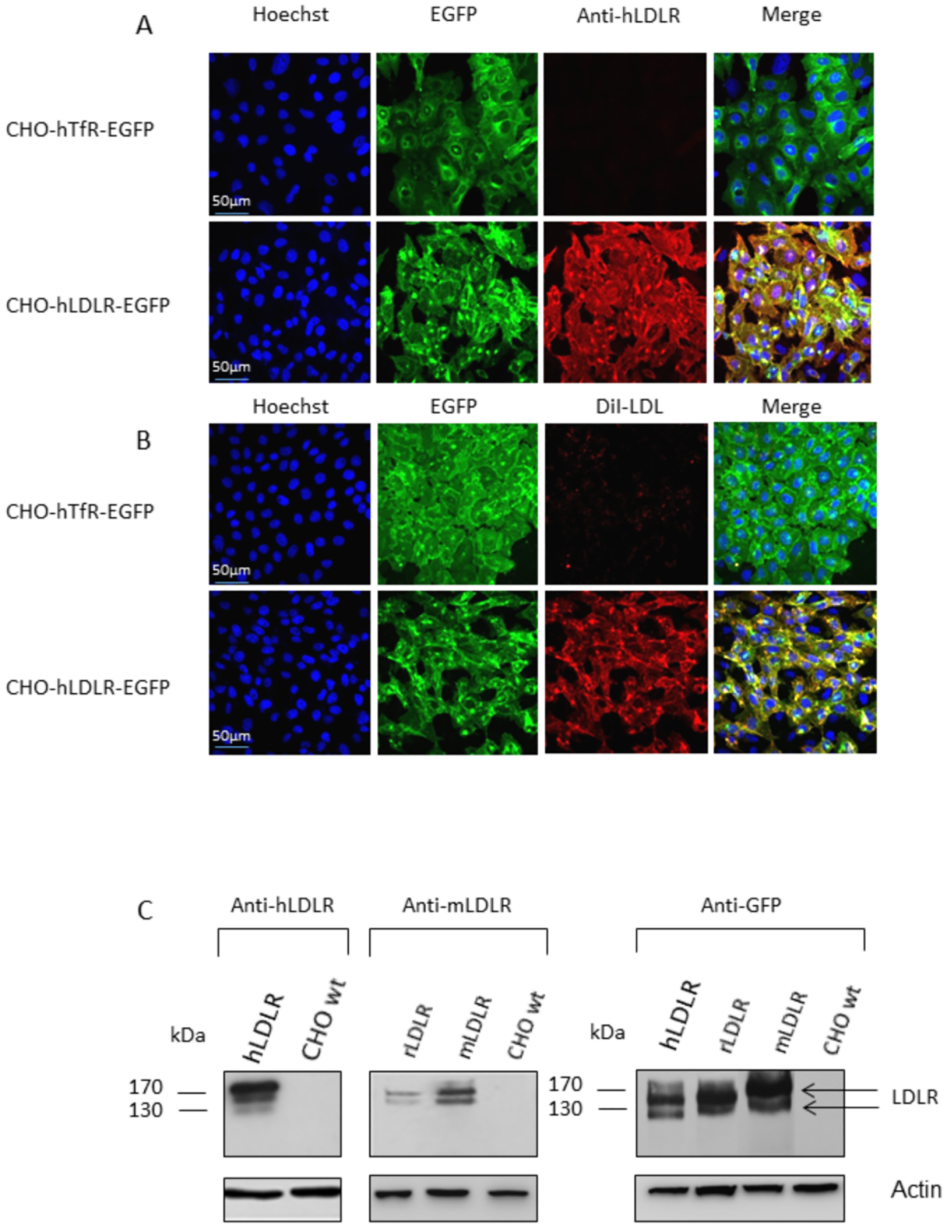
Membrane expression and functionality of hLDLR expressed by the CHO-hLDLR-EGFP cell line. (A) Representative confocal photomicrographs of immunostained CHO-hTfR-EGFP (negative control) and CHO-hLDLR-EGFP cells (green) with an anti-LDLR antibody diluted at 1/100 (red) following triton X100 permeabilization of the cell membrane. Cell nuclei are labeled with Hoechst#33258 at 0.5 μg/mL (blue). Co-labeling appears in yellow in the merged pictures. Only the cells with stable expression of the hLDLR-EGFP construct express the membrane receptor. (B) Representative confocal photomicrographs of CHO-hTfR-EGFP and CHO-hLDLR-EGFP cells (green) after a 10 min incubation period with DiI-LDL 15 μg/mL (red). Cell nuclei are labeled with Hoechst#33258 (blue). Co-labeling appears in yellow in the merged pictures. DiI-LDL is essentially bound to the CHO-hLDLR-EGFP cells indicating that the hLDLR-EGFP chimera receptor binds its ligand. (C) Western blots performed on cell membrane preparations of CHO cells expressing hLDLR-EGFP, rLDLR-EGFP and mLDLR-EGFP compared to CHO WT, using anti-hLDLR (1/800) and anti-rat and mouse LDLR antibodies (1/1000). These bands are also labeled with an anti-EGFP antibody. Bands of 140 kDa and 190 kDa correspond respectively to the immature and mature LDLR-EGFP fusion proteins (arrows). Actin was used to check loading of equal amounts of protein.

Full-length coding regions for hLDLR, for mouse LDLR (mLDLR) and for rat LDLR (rLDLR) were amplified by RT-PCR from brain total RNA. The amplified cDNAs were cloned in frame with EGFP in pEGFP-N1 vector. We generated stable cell lines in CHO cells with hLDLR and mLDLR constructs, including an EGFP control cell line. A cell line with stable expression of the human transferrin receptor (hTfR) fused to EGFP was also used as alternative control. Cell lines were referred to as CHO-hLDLR-EGFP; CHO-mLDLR-EGFP; CHO-EGFP; CHO-hTfR-EGFP. We checked constitutive plasma membrane expression of the hLDLR and mLDLR fused to EGFP by immunocytochemistry with antibodies against the hLDLR (Fig 1A) by binding assays of fluorescent DiI-LDL on the cell lines (Fig 1B), and by western blot using different antibodies that detect preferentially the LDLR from one or several species (Fig 1C). The LDLR-EGFP appears as 180 and 140 kDa bands in all species, presumably resulting from partial glycosylation rather than cleavage of the EGFP moiety, considering that both bands are also detected with an anti-EGFP antibody.

Different phage display libraries presenting linear or cyclic constrained peptides (Cys residue at both extremities of the peptides) on pIII or pVIII proteins of M13 or fd phage were used for biopanning (4 to 5 rounds) on the stable cell line expressing the hLDLR-EGFP, following subtraction of the libraries on the control CHO-EGFP cells. A total of 7 independent screens were performed with the different libraries. Several hundred phage clones were sequenced which allowed selection of 28 distinct peptide sequences with 9 to 15 residues that were divided into 2 families following ClustalW sequence alignment: 17 linear peptides with limited sequence similarities (one sequence, VH549 shown as an example, Table 2) and 11 cyclic peptides with a consensus Met-Pro-Arg (MPR) motif flanked by 2 Cys residues (Table 2).

**Table 2:** Sequences of LDLR targeting peptides presented by phage.

Peptide	Sequence
VH101	HLDCMPRGCFRN
VH202	CQVKSMPRC
VH203	CTTPMPRLC
VH204	CKAPQMPRC
VH205	CLNPSMPRC
VH306	CLVSSMPRC
VH307	CLQPMPRLC
VH308	CPVSSMPRC
VH309	CQSPMPRLC
VH310	CLTPMPRLC
VH411	DSGLCMPRLRGCDPR
Consensus	(X)_n_C(X)_n_MPR(X) _n_C(X)_n_
VH549	TPSAHAMALQSLSVG

(X)_n_ in the consensus sequence corresponds to 0 to 4 amino-acid residues. The underlined sequences between the 2 cysteines in the 11 first peptides are presumably cyclic. The VH549 peptide is linear.

**Fig 2:**
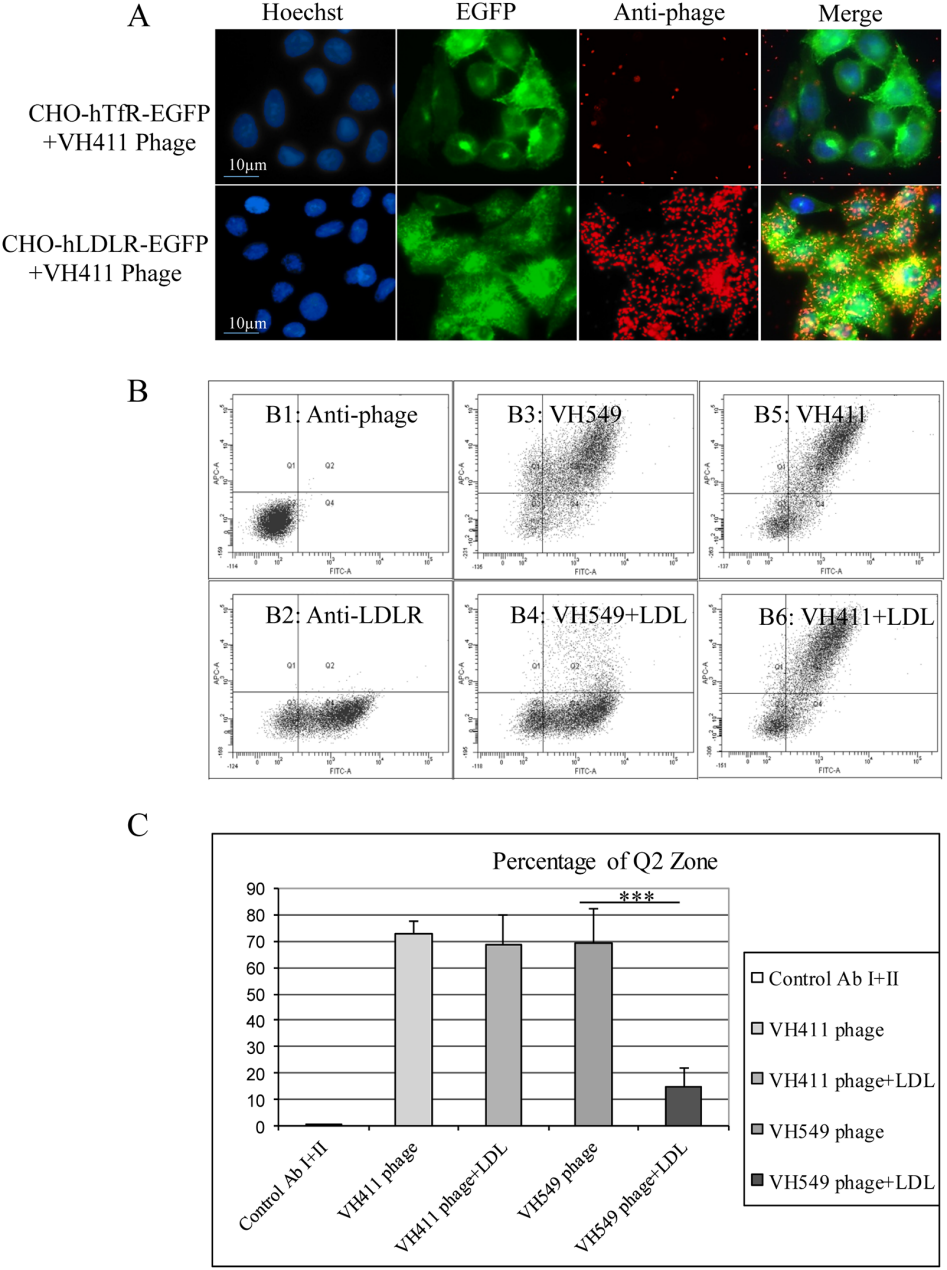
*In vitro* validation of VH411 phage binding. (A) Representative epifluorescence photomicrographs of CHO-hTfR-EGFP and CHO-hLDLR-EGFP cells (green) incubated with 1.10^10^ VH411 fd phage immunodetected with an anti-PVIII antibody diluted at 1/3000 (red). Cell nuclei are labeled with Hoechst#33258 at 0.5μg/mL(blue). Co-labeling appears in yellow/orange in the merged pictures. fd phage do not bind on cells that express the hTfR-EGFP. (B) LDLR expression and binding of phage with affinity for LDLR in the absence or presence of LDL at 0.5 mg/mL was evaluated by FACS on HUVEC for 20 min at 4°C in order to avoid endocytosis. In the present example, the cells were incubated with the anti-PVIII antibody (1/1000) detected with an allophycocyanine (APC) secondary antibody (1/800) (vertical axis) (B1, B3, B4, B5, B6) and with the anti-LDLR antibody (1/50) detected with a secondary FITC-labeled antibody (1/800) (horizontal axis) (B2, B3, B4, B5, B6). Phage VH549 (linear) and VH411 (cyclic) were added to cells in the absence (B3, B5 respectively) or presence of LDL (B4, B6 respectively). The number of positive cells was standardized with 5,000 events for each test. The results were expressed in arbitrary units of fluorescence. LDL strongly decreases the binding of VH549 but has no impact on VH411 binding. (C) Quantification of the fluorescence signal in zone Q2 of the graphs in B. No shift is measured in Q2 for the VH411 phage in the presence of LDL while a 56% reduction in Q2 signal is measured for VH549 phage with LDL indicating competition for the LDL binding site. Statistical analysis was performed using an analysis of variance, followed by Student’s test. Values represent the mean of 3 independent experiments; ***p ≤ 0.001.

Phage clones were individually checked for selective binding to the CHO-hLDLR-EGFP but not to the CHO-EGFP or hTfR-EGFP control cell lines. For all 29 peptides, we observed correlation between hLDLR-EGFP localization and phage immunostaining with anti-M13 bacteriophage antibody as exemplified for phage VH411 (Fig 2A). Similar experiments were performed on mouse and rat CHO-LDLR-EGFP cell lines in order to guarantee that peptides to be developed as targeting moieties for the hLDLR could undergo preclinical validation in rodents.

Next, we performed a competition assay between individual phage clones in order to determine which peptides presented the highest affinity for the target receptor: the same number of phage (2.10^10^) from individual clones encoding cyclic peptides on the one hand and linear peptides on the other were mixed and incubated on the CHO-hLDLR-EGFP cell line. Following extensive wash and phage elution, 40 individual phage plaques for each family of phage clones were picked and sequenced. Among the cyclic and linear peptide families, VH411 and VH549 were the most efficient in displacing other phage: 80% and 38% of the sequences from the phage encoding the cyclic and linear peptides respectively corresponded to the VH411 and VH549 phage clones, which were selected for further studies. Using flow cytometry we next assessed whether the VH411 and VH549 phage clones competed with LDL upon binding to the hLDLR. We used human umbilical vein endothelial cells (HUVEC) as primary cells of human origin that constitutively express physiological levels of the hLDLR. Cells were incubated in the presence of 1.10^10^ phage and LDL and phage bound to the HUVEC were assessed by flow cytometry with a mouse anti-M13 bacteriophage antibody. Correlation with hLDLR expression levels was assessed in the same experiments using a rabbit anti-LDLR antibody (Fig 2B) and quantification is summarized in (Fig 2C). Phage encoding cyclic peptides do not compete with LDL binding, while those encoding linear peptides do as shown with the VH411 and VH549 phage respectively. For this reason, and because cyclic peptides are *a priori* more stable than linear peptides, we pursued peptide-vector development based on the peptide sequence displayed by the VH411 phage.

### Characterization of VH411 peptide binding to hLDLR and peptide optimization

**Fig 3:**
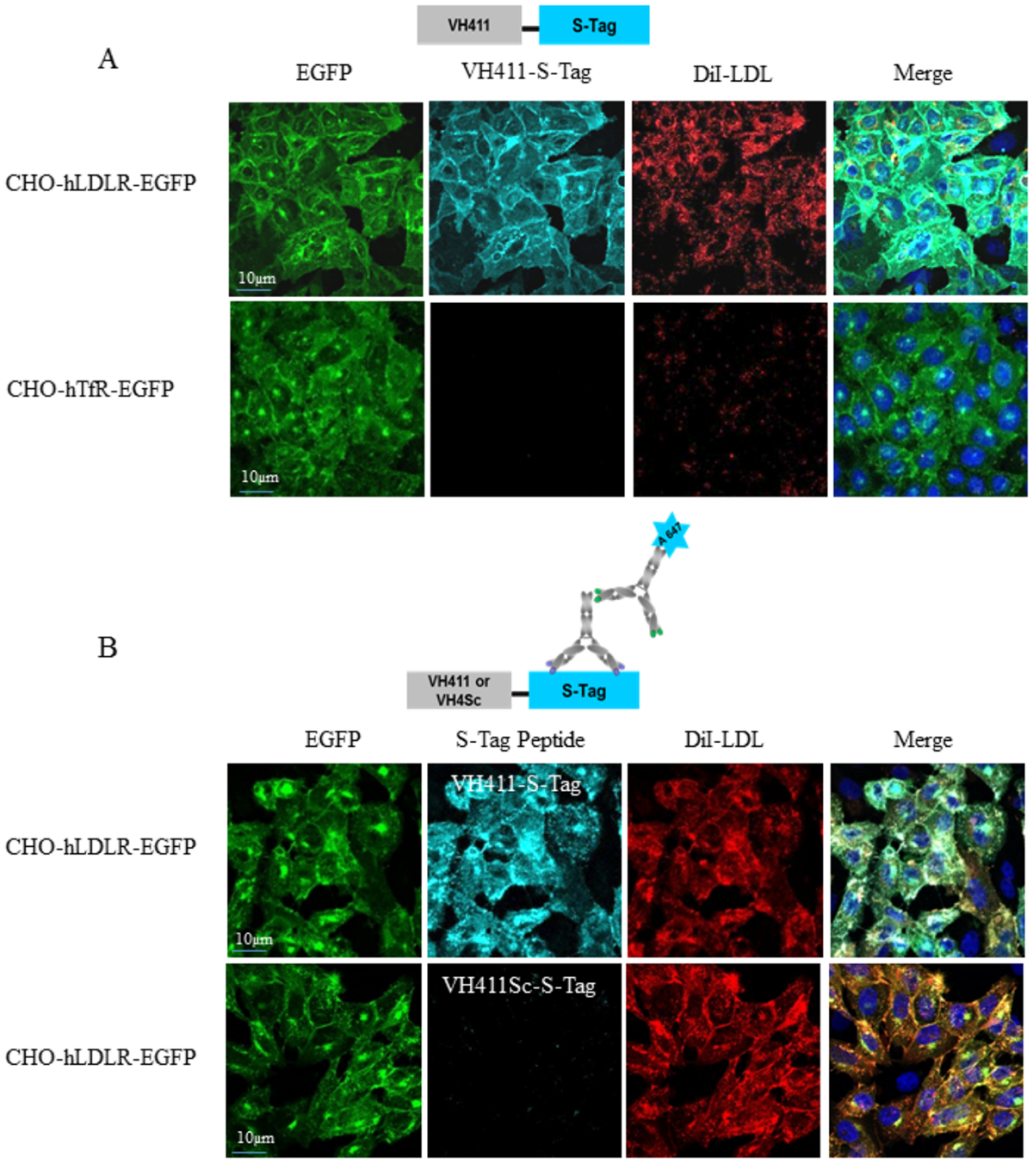
Characterization of VH411-S-Tag peptide conjugate binding to the CHO-hLDLR-EGFP cell line. (A) Representative confocal photomicrographs of CHO-hLDLR-EGFP and CHO-hTfR-EGFP cells (green) incubated 1 hr at 37°C with 10 μM of VH411-S-Tag detected post-fixation with an anti-S-Tag and A647-conjugated secondary antibody (cyan) and 10 μg/mL of DiI-LDL. Cell nuclei are labeled with Hoechst#33258 (blue) at 0.5 μg/mL. Co-labeling appears in blue-green/orange in the merged pictures. Note that VH411-S-Tag co-localizes with hLDLR and is internalized by CHO-hLDLR-EGFP cells. (B) Representative confocal photomicrographs of CHO-hLDLR-EGFP cells (green) incubated 1 hr at 37°C with a macromolecular complex (see scheme Fig 3B) resulting from the co-incubation and interaction of 10 μM VH411-S-Tag peptide or VH411Sc-S-Tag peptide with the anti-S-Tag antibody (1/200) and the Alexafluor 647-conjugated secondary antibody (1/800) (cyan). Cells were concomitantly exposed to 10 μg/mL of DiI-LDL (red). Cell nuclei are labeled with Hoechst#33258 (blue). Co-labeling appears in blue-green/orange in the merged pictures.

We synthesized the 15-residue VH411 peptide (DSGLCMPRLRGCDPR) and a scrambled version of this peptide, VH411Sc (RDRMCGRDLPSCGPL), both conjugated in C-ter to a 3 glycine spacer and a 15 mer S-Tag peptide (KETAAAKFERQHMNS) [22] to respectively generate VH411-S-Tag and VH411Sc-S-Tag peptides. One of the properties of the S-Tag is its detection by an anti S-Tag antibody for immunocytochemistry. The VH411-S-Tag conjugate bound the CHO-hLDLR-EGFP cells (Fig 3A) while no binding was observed on the CHO-hTfR-EGFP (Fig 3A) or CHO-EGFP control cell lines. We next incubated CHO-EGFP and CHO-hLDLR-EGFP cells with VH411-S-Tag or VH411Sc-S-Tag complexed with a primary anti-S-Tag antibody and a secondary fluorescent Alexa594-coupled antibody (see scheme Fig 3B). Confocal microscopy analysis showed that only the VH411-S-Tag peptide elicited binding of the macromolecular complex onto the CHO-hLDLR-EGFP cells, and endocytosis (Fig 3B).

Using Surface Plasmon Resonance (SPR), we showed that affinity of VH411-S-Tag for recombinant hLDLR extracellular domain was in the same range than the non-tagged VH411 peptide with affinities of 448 nM versus 250 nM respectively (Table 3). We thus provided evidence that the VH411 peptide had the potential to be further developed as a peptide-vector for transport of payloads into cells expressing the hLDLR. Because VH411 is a rather large peptide encompassing only natural amino acid residues, we aimed to improve its intrinsic properties by a medicinal chemistry-based optimization to reach good prerequisites for further preclinical development.

**Table 3:** Sequences and K_D_ of LDLR targeting peptides, free or conjugated/fused to different molecules.

Peptide/conjugate	Sequence ^a^	*k*_on_ (M^-1^s^-1^)	*k*_off_ (s^-1^)	*K*_D_ (nM)
**Peptide vectors**				
VH411	Ac-DSGLCMPRLRGCDPR-NH_2_	b	b	250 (±9)
VH434	Pr-CMPRLRGC-NH_2_	b	b	196 (±33)
		2,3 (±0,9).10^5^	6,4(±2,8).10^−2^	280 (±33)^c^
VH445	Pr-cMPRLRGC-NH_2_	b	b	76 (±17)
		6,79 (±0,5).10^5^	1,59 (±0,2).10^−1^	233 (±24)^c^
VH4127	Pr-cMThzRLRG"Pen"-NH_2_	b	b	18 (±5)
		b	b	36 (±1)^c^
**Conjugates**				
VH411-S-Tag	Ac-DSGLCMPRLRGCDPR-GGG-KETAAAKFERQHMDS-NH_2_	2.1(±0,2).10^5^	9.4(±2,7).10^−2^	448 (±114)
VH445-S-Tag	Pr-cMPRLRGC-GGG-KETAAAKFERQHMDS-NH_2_	b	b	92 (±17))
VH4127-S-Tag	Pr-cM"Thz"RLRG"Pen"-GGG-KETAAAKFERQHMDS-NH_2_	b)	b	19 (±4)
Cy5.5-PEG6-VH4127	Cy5.5-PEG6-cM"Thz"RLRG"Pen"-NH_2_	1.3 (±0.6).10^6^	7.7 (±2.9).10^−2^	61 (±6)
(VH434)_2_-Fc	hIgG1Fc-GGG-(CMPRLRGC)_2_	2.6 (±0.5).10^5^	2.8 (±1).10^−4^	1.2 (±0,5)
(VH04sc)_2_-Fc	hIgG1Fc-SMCC- (Pr- cRPLGRMC-G-(CH_2_)_2_-S)_2_	-	-	-
(VH4127)_2_-Fc	hIgG1Fc-SMCC-(Pr-cM"Thz"RLRG"Pen"-G-(CH_2_)_2_-S)_2_	4.7 (±3).10^6^	1.3 (±0.3).10^−4^	0.04 (±0.03)
SiGLO CyclophilineB-VH4127	SiGLOCycloB-MCC-(Pr-cM"Thz"RLRG"Pen"-G-(CH_2_)_2_-S)	1.7 (±1,3).10^5^	3.1 (±0.7).10^−2^	250 (±129)
**Dimer**				
VH445 Dimer	(Pr-cMPRLRGC-G-NH-CH_2_-CH_2_)_2_-N-(CH_2_)_2_-NH_2_	2 (±3).10^6^	6.2 (±3.3).10^−3^	13 (±10)
VH4127 Dimer	(Pr-cM"Thz"RLRG"Pen"-G-NH-CH_2_-CH_2_)_2_-N-(CH_2_)_2_-NH_2_	3.3 (±2).10^6^	1 (±0.8).10^−2^	2.9 (±0.7)

^(a)^ One-letter amino acid code; otherwise specified (non-natural amino acids), lower case letters indicate (D)-configuration. “Ac-” and “Pr-” mean N-terminal acetylation and propionylation, respectively.

^(b)^ *k*_on_ and *k*_off_ details in (Jacquot et al., 2016). The underlined sequences refer to a disulfide bridge between the 2 cysteines or analogues.

^(c)^ Measurements were performed on the immobilized murine LDLR (mLDLR). (-) No binding. All kinetic parameters (*k*_on_ and *k*_off_) and *K*_D_ values are measured using the Langmuir 1:1 model except for VH445 and VH4127 dimers where a bivalent analyte model was applied. In this case, kinetic parameters and *K*_D_ values of the first component of the bivalent analyte interaction are mentioned. Sensorgrams for peptides/conjugates are provided in S2 Fig.

We optimized VH411 as described previously in [23]. Successive truncations of the N- and C-termini of VH411 led to the shortened 8-residue VH434 peptide (CMPRLRGC, referred to as peptide 19 in [23] with no loss of binding on hLDLR and mLDLR (K_D_ = 196 nM and 280 nM respectively, Table 3). We have shown with linear peptides whose cysteines were methylated or replaced by serines [23] that the bridge between cysteines (or analogs of cysteines containing a thiol moiety) is necessary for peptide binding to the LDLR. A D-amino acid scan identified a lead peptide (VH445, cMPRLRGC) containing a (D)-Cys in position 1 with a 3.3 fold-increased affinity compared to VH411 as assessed by SPR (Table 3). We performed further chemical optimization of VH445 as detailed elsewhere [24] that led to a series of novel peptides, in particular VH4127 cMThzRLRGPen encompassing a D-Cysteine at the first amino acid position and two non-natural amino acids, namely thiazolidine-4-carboxylic acid (Thz) and penicillamine (Pen) instead of the initial proline and cysteine at positions 3 and 8, respectively (Table 3).

### Characterization of the hLDLR domain bound by the selected peptides

**Fig 4:**
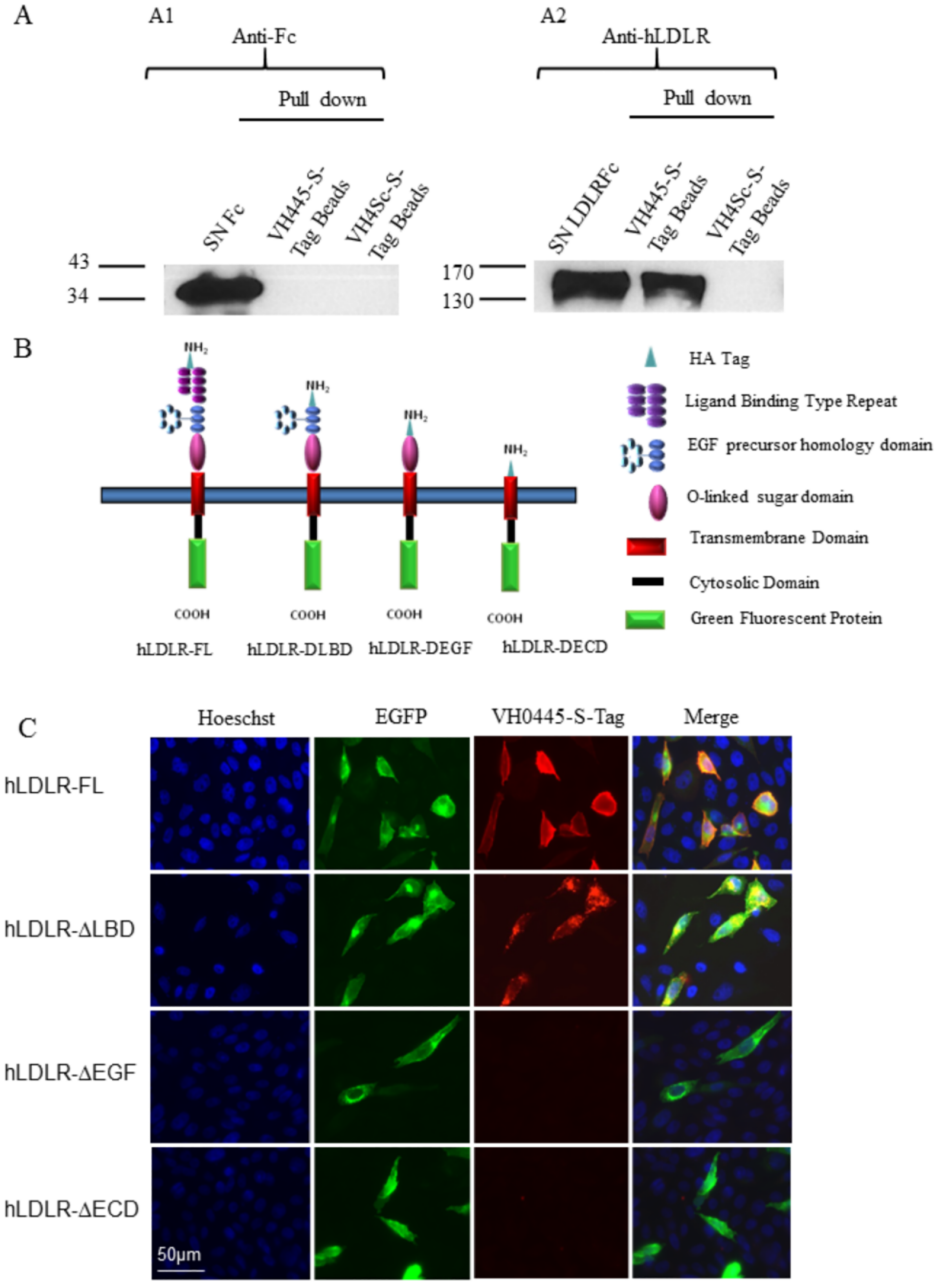
Identification of the hLDLR domain that interacts with VH0445-S-Tag. (A) A pull-down assay was performed in concentrated supernatant of HEK cells transiently transfected with p-Fc as control or with phLDLR-Fc vector, using epoxy beads coated with VH0445-S-Tag or with VH4Sc-S-Tag control peptide (scrambled version of VH445). Western blots were carried out using anti-Fc (A1) and anti-hLDLR antibodies (A2). Note that the hLDLR is pulled-down by the beads coated with the VH0445-S-Tag peptide but not with the VH4Sc-S-Tag peptide. (B) Schematic representation of truncated LDLR (hLDLR-FL, hLDLR-ΔLBD, hLDLR-ΔEGF and hLDLR-ΔECD, all encompassing a HA Tag to verify appropriate extracellular distribution of the extracellular domains) produced by cells transfected with different plasmid constructs derived from hLDLR-pEGFP. (C) VH445-S-Tag binding to the hLDLR EGF precursor homology domain. Representative fluorescence micrographs of CHO cells transiently expressing hLDLR-FL, hLDLR-ΔLBD, hLDLR-ΔEGF and hLDLR-ΔECD (all in green) incubated with 10 μM of VH0445-S-Tag, goat Anti-S-Tag (1/200) and anti-goat Alexa 594 (1/800) (red). Cell nuclei are labeled with Hoechst#33258 (blue). Co-labeling appears in yellow/orange in the merged pictures.

In order to exclude that the peptide family we characterized bound an irrelevant protein co-expressed with the hLDLR in the CHO-hLDLR-EGFP cell line, we performed a pull-down assay using the VH445 peptide. The extracellular domain of the hLDLR was expressed in fusion with a human IgG1-Fc fragment (hLDLR-Fc) in HEK293 cells. The secreted hLDLR-Fc and Fc fragment alone, used as control, were applied to epoxy magnetic beads coated with VH445-S-Tag or with VH4Sc-S-Tag, encompassing a scrambled version of VH445 (VH4Sc peptide: cRMLGRPC). Proteins retained by the coated beads were analyzed by western blot using an anti-hLDLR antibody. hLDLR-Fc bound efficiently to beads coated with VH445-S-Tag but not VH4Sc-S-Tag, while the Fc alone bound to neither peptide (Fig 4A).

The extracellular hLDLR encompasses several functional domains: the ligand binding domain (LBD), the EGF precursor homology domain and the O-glycosylation rich domain. In order to determine which of these domains binds the VH445 peptide, we removed sequentially each of them and expressed the resulting “mini receptors” in transfected CHO cells (scheme in Fig 4B). Binding of the VH445-S-Tag was assessed on these transfected cells with an anti-S-Tag antibody. The VH445 did not bind the LBD, consistent with the absence of competition of the natural ligand LDL with the VH411-displaying phage (see Fig 2B and 2C). Instead, the VH445 bound the EGF precursor homology domain, which encompasses the EGF A, EGF B, beta propeller and EGF C domains (Fig 4C).

### *In vitro* binding properties of VH445 and VH4127

The affinity (K_D_) of VH445 and VH4127 for the hLDLR ectodomain, as assessed using SPR, was measured at 76 and 18 nM, respectively (Table 3). VH445 and VH4127 affinity for mouse LDLR extracellular domain was in the same range than for hLDLR (K_D_ = 233 nM and 36 nM respectively). It has been described that dimerized or oligomerized ligands can exhibit higher affinity constants for their target receptor [25, 26]. We thus synthetized dimers of VH445 and VH4127 peptides. Each dimer was synthesized by coupling two copies of each peptide on a tripodal triamine commercial platform (TREN: tris(2-alkyl-2-aminoethyl)amine), the last arm of the platform being free for further functionalization with a reporter or a therapeutic agent of interest. Interestingly, using SPR, we observed a clear increase in the affinity of both peptide dimers (see S2 Fig, last column) for the hLDLR with a very significant modification of the interaction profiles compared to VH445 and VH4127 monomers (S2 Fig, first column). As the dissociation phase was too slow to be accurately determined using a Langmuir 1:1 model, the binding data were fitted using a bivalent analyte model. K_D_ values of the first (monovalent) binding step for peptide dimers were reported in Table 3. These values cannot be directly compared to those obtained for monomeric peptides (using Langmuir 1:1 model) but we observe that the relative affinity value between VH4127 and VH445 dimers is the same than between VH445 and VH4127 monomers.

### Potential of the peptide-vectors to induce LDLR-dependent uptake of different cargos

#### Conjugation of peptide-vectors to fluorophores

**Fig 5:**
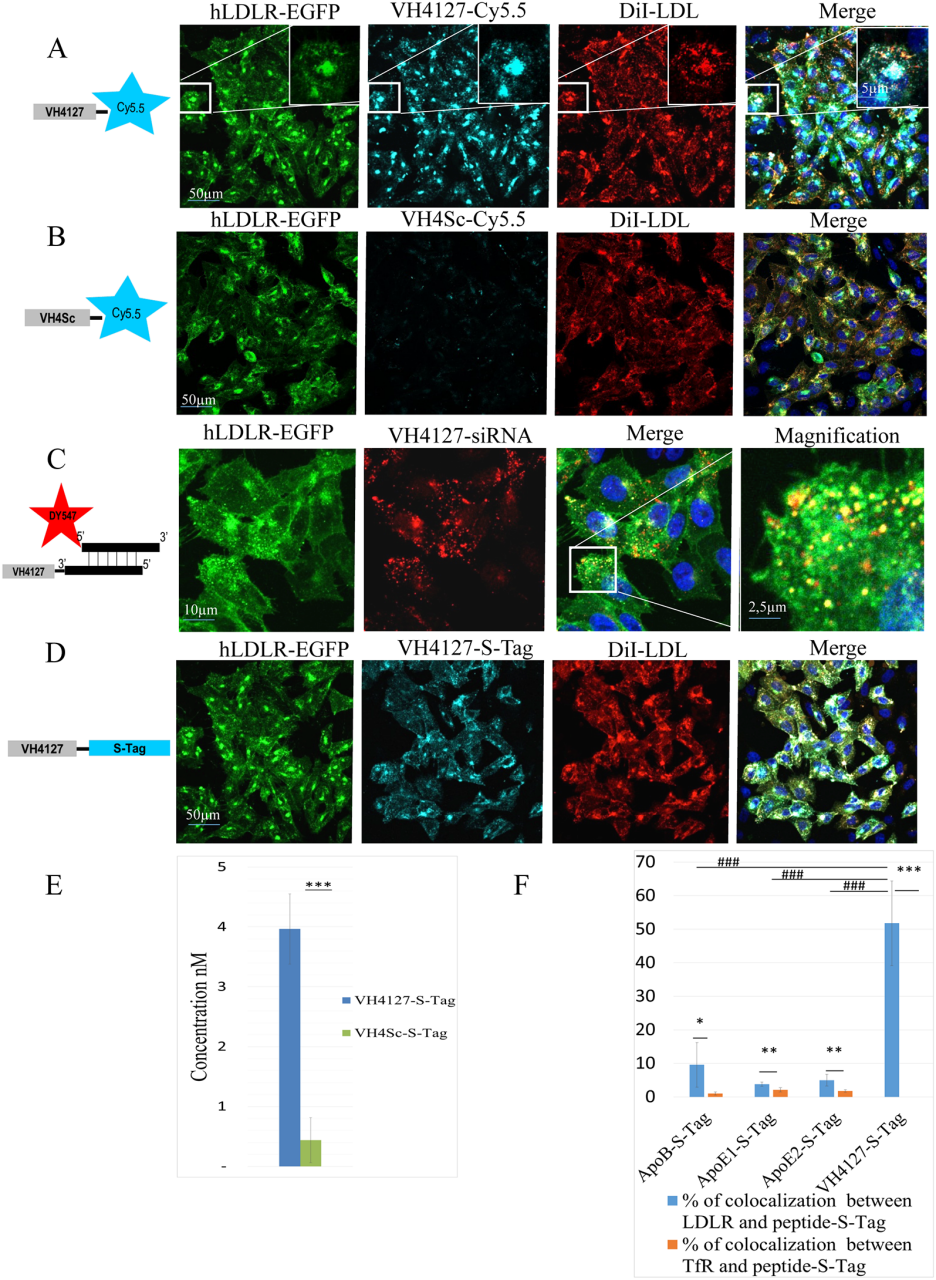
Interaction of VH4127 conjugates with hLDLR and comparison with ApoB and ApoE peptides. Representative confocal fluorescence micrographs of CHO-hLDLR-GFP cells incubated with 10 μM of VH4127-Cy5.5 (light blue) and 20μg/mL of DiI-LDL (red). Insets correspond to higher magnification of boxed cells. (B) CHO-hLDLR-GFP cells incubated with 10 μM of VH4Sc-Cy5.5 and 20μg/mL of DiI-LDL (red). (C) CHO-hLDLR-GFP cells incubated with 10μM of VH4127-siGLO Cyclophiline B (red). (D) CHO-hLDLR-GFP cells incubated with 10μM of VH4127-S-Tag (light blue) following S-Tag detection with a goat anti-S-Tag and a secondary A647-conjugated anti-Goat antibody. Cell nuclei are labeled with Hoechst#33258 (blue). Co-labeling appears in yellow in the merged pictures. Note that all conjugates bind and largely co-localize with LDLR. (E) ELISA quantification of bound/endocytosed VH4127-S-Tag or VH4Sc-S-Tag conjugates to CHO-hLDLR-GFP cells 1 hr post-incubation at 10μM. The graph represents the mean of concentration in nM ± SD (n = 3 per conjugate, *p ≤ 0.05, **p ≤ 0.01, ***p ≤ 0.001). (F) Image J quantification of the percentage of co-localization ± SD (n = 3 per peptide) between hLDLR-GFP (blue graph bars) or hTfR-GFP (orange graph bars) and ApoB, ApoE1, ApoE2 and VH4127 peptides conjugated to the S-Tag (*p ≤ 0.05, **p ≤ 0.01, ***p ≤ 0.001). The percentage of co-localization on the hLDLR-GFP (blue graph bars) between the ApoB, ApoE1 and ApoE2 peptides conjugated to the S-Tag was also compared to that of VH4127-S-Tag (### p ≤ 0.001).

As models for small organic molecules, probes varying from hydrophobic molecules such as Cyanins, to charged and hydrophilic dyes such as sulfated Cyanin5.5 or Alexa dyes were conjugated to the VH445 and/or VH4127 peptides. We evaluated the influence of the C-ter or N-ter conjugation of the peptide-vectors to these molecules on the binding affinity to the hLDLR. We used linkers of different nature and size and concluded that our peptide-vectors can be functionalized without significant loss of binding affinity for their target receptor. Peptides conjugated to RhoRedX, Cy3.5 and Cy5.5 were evaluated for binding and endocytosis on the CHO-hLDLR-EGFP cell line. One example of the typical results observed with these fluorophores is shown with the VH4127 or the control VH4Sc peptides conjugated on their N-ter to Cy5.5 via a PEG linker (Fig 5A and 5B respectively). SPR analysis showed that the Cy5.5-PEG6-VH4127 conjugate retained affinity for hLDLR despite a slight 3-fold increase in K_D_ (Table 3). Confocal microscopy analysis showed that only the fluorophore-conjugated peptide that targets LDLR readily bound and was internalized in an LDLR-dependent manner as indicated by pulse chase experiments showing DiI-LDL co-localization with LysoTracker or Cy5.5-PEG6-VH4127 (detailed in S3 Fig).

#### Conjugation of peptide-vectors to siRNAs

We assessed the potential of the peptide-vectors to be conjugated to siRNAs. A siGLO cyclophilinB siRNA labeled with DY547 on the 5’end of its sense strand was conjugated to VH4127 and to its control scrambled peptide VH4Sc using a heterobifunctional linker (sulfo-SMCC) conjugated to an aminohexyl moiety introduced on the 3′-terminus of the siRNA antisense strand. Both purified cyclophilinB siRNA conjugates were tested on the hLDLR-EGFP cell line. The VH4127-siRNA conjugate bound the hLDLR expressing cell line and was endocytosed (Fig 5C) while there was no binding of the VH4Sc-siRNA. SPR analysis showed that this conjugate retained binding affinity for hLDLR despite a 10-fold decrease of the K_D_ value compared to the unconjugated peptide (Table 3).

#### Conjugation of peptide-vectors to peptides

VH445 and VH4127 were conjugated in tandem to the S-Tag peptide as described above for VH411 validation and we showed by SPR that S-Tag conjugation did not change the kinetic parameters and thus affinity for hLDLR (Table 3). Overlap between hLDLR-EGFP and VH4127-S-Tag cellular distribution confirmed that binding and internalization of this S-Tag-conjugated peptide was LDLR-dependent (Fig 5D). Using an ELISA assay based on S-Tag detection set-up in-house we showed that following 1 hr incubation at 37°C, there was a ~10-fold difference in VH4127-S-Tag conjugate binding/accumulation compared to VH4Sc-S-Tag (Fig 5E).

We next compared the LDLR-mediated uptake of VH4127 with other reported LDLR-binding peptides ApoB, ApoE1 and ApoE2 [27–30]. All peptides were conjugated to the S-Tag peptide as reporter. We used Image J software to assess the percentage of co-localization of the different S-Tag-peptides with hLDLR and hTfR (control) expressed by the CHO-hLDLR-GFP and CHO-hTfR-GFP cell lines, respectively. At the same incubation concentration, the percentage of hLDLR that co-localized with VH4127-S-Tag was over 50% while it was under 10% for ApoB-S-Tag, ApoE1-S-Tag and ApoE2-S-Tag (Fig 5F). No co-localization with hTfR was quantified for VH4127-S-Tag, showing the specificity of the LDLR binding.

#### Conjugation of peptide-vectors to an antibody constant fragment

**Fig 6:**
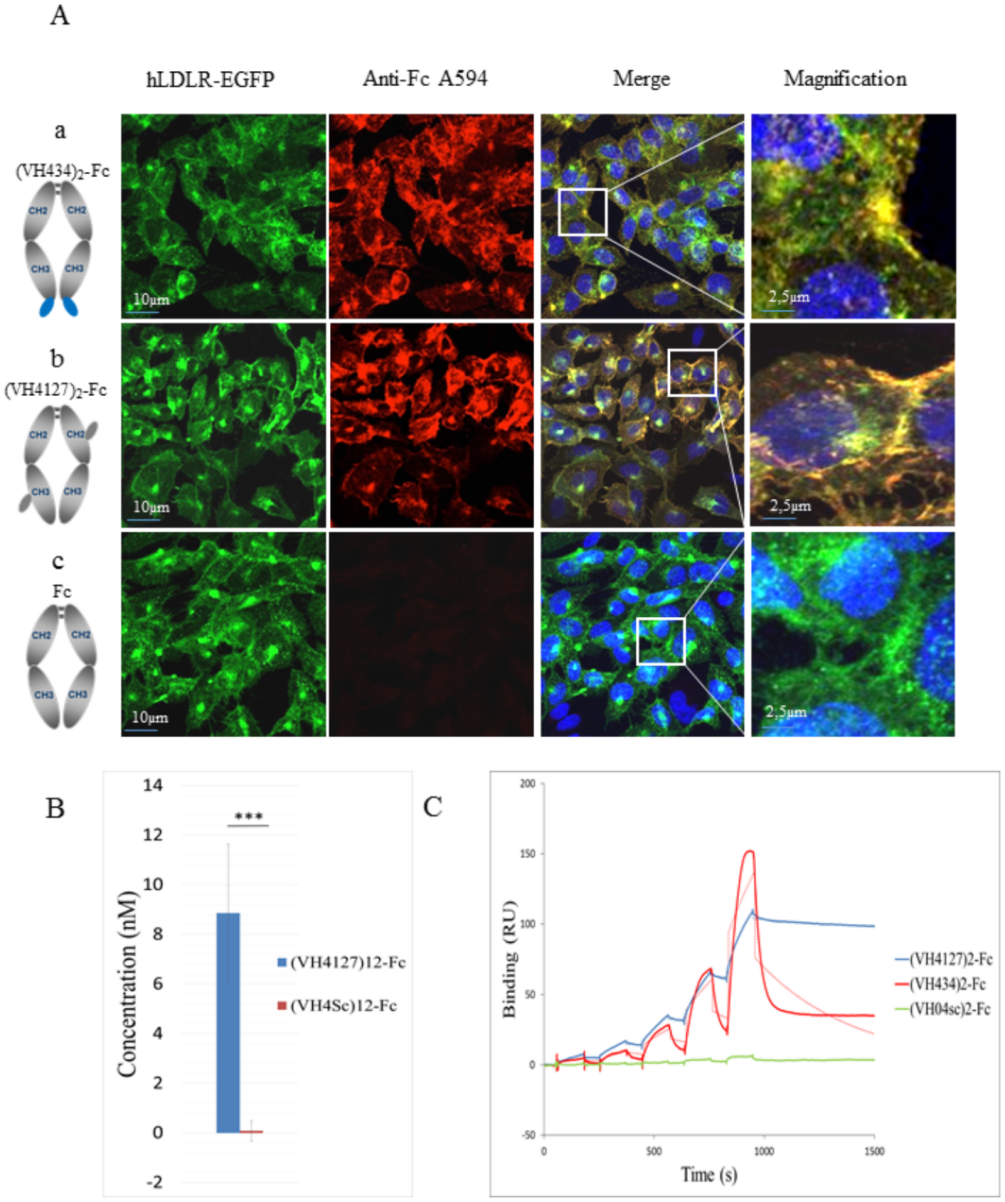
Binding of (VH434)_2_-Fc, (VH4127)_2_-Fc, Fc and (VH4Sc)_2_-Fc to the hLDLR. Representative confocal fluorescence micrograph of CHO-hLDLR-GFP cells incubated with 10 nM of (VH434)_2_-Fc (a), (VH4127)_2_-Fc (b) and Fc alone (c) used as a negative control. The Fc and Fc-fusion/conjugates were detected with an anti-hFc Alexa 594-conjugated antibody (red). Co-labeling of LDLR-GFP (green) and Fc fusion/conjugates appear in yellow in the merged pictures, as evidenced at higher magnification. (B) ELISA quantification of bound/endocytosed (VH4127)_2_-Fc or (VH4Sc)_2_-Fc conjugates to CHO-hLDLR-GFP cells 1 hr post-incubation at 10 nM (n = 3 per conjugate; ***p ≤ 0.001). Note that (VH4127)_2_-Fc binding/endocytosis to the CHO-hLDLR-GFP cells is increased ≈300-fold compared to the (VH4Sc)_2_-Fc control. (C) Representative sensorgrams of Fc-fusion/conjugate binding to the extracellular domain of hLDLR. A set of concentrations (0.5–80 nM for the Fc-conjugates or 5–80 nM for the Fc-fusion) was sequentially injected over immobilized hLDLR. The solid lines represent the specific binding of each Fc-fusion/conjugate and the dotted lines represent the fit of the data obtained with a Langmuir 1:1 model.

Finally, we conjugated the peptide-vectors to an antibody fragment to assess their ability to carry larger cargos such as proteins into cells upon direct fusion or chemical conjugation of the peptide-vectors. The fragment crystallizable region (Fc) of a human IgG1 antibody was used as a prototypic protein. Two types of conjugates were evaluated: i) we generated a recombinant Fc fragment fused in N- or C-ter with VH434, the all-natural amino acid peptide version of VH445; because of Fc fragment dimerization, this fusion conjugate has a valency of 2 VH434 peptides per dimerized Fc, referred to as (VH434)_2_-Fc; ii) we chemically conjugated in a random fashion VH4127 and VH4Sc peptides encompassing a thiol moiety to lysine side chains of the Fc fragment using heterobifunctional linkers such as amine-to-thiol coupling reagent (i.e sulfo-SMCC). Upon chemical conjugation we quantified by MALDI-TOF mass spectrometry a mean ratio of 2 peptides per dimeric Fc fragment, referred to as (VH4127)_2_-Fc.(see Table 3 for affinity data). We first assessed binding of the Fc conjugates on the hLDLR by immunocytochemistry: the (VH434)_2_-Fc fusion (Fig 6A, panels a) and (VH4127)_2_-Fc chemical conjugates (Fig 6A, panels b) and their controls, non-conjugated Fc (Fig 6A, panels c) or conjugated to the scrambled VH4Sc peptide were incubated at various concentrations (from 100 nM to 0.1 nM) on the CHO-hLDLR-EGFP and CHO-WT cell lines. We observed that the different peptide-vectors, whether in fusion or chemically conjugated, promoted efficient binding of the Fc fragment to the hLDLR-EGFP and endocytosis, and that there was a correlation between the LDLR expression levels and uptake of vectorized Fc. No uptake was observed in the same conditions with the Fc fragment alone or control (VH4Sc)_2_-Fc conjugate. (VH4127)_2_-Fc binding/endocytosis on the CHO-hLDLR-EGFP cell line was more than 300-fold higher than (VH4Sc)_2_-Fc as quantified by anti-Fc ELISA (Fig 6B). The equilibrium dissociation constants of (VH434)_2_-Fc and (VH4127)_2_-Fc for the hLDLR extracellular domain were estimated using SPR at 1.2 and 0.04 nM, respectively, using a Langmuir 1:1 model (Fig 6C and Table 3). These decreased K_D_ values compared to the K_D_ values of the free peptides most likely resulted from an avidity effect due to the peptide-vector ratio per dimerized Fc Fragment (2 for VH434-Fc conjugate and 2 for the VH4127-Fc conjugates). Indeed, as for the VH445 and VH4127 dimers, this affinity increase was mainly due to the decrease of the dissociation rate constant (k_off_), while association rate constants remained similar to free peptide-vectors.

### *In vivo* LDLR targeting of the vectorized Fc fragment

**Fig 7:**
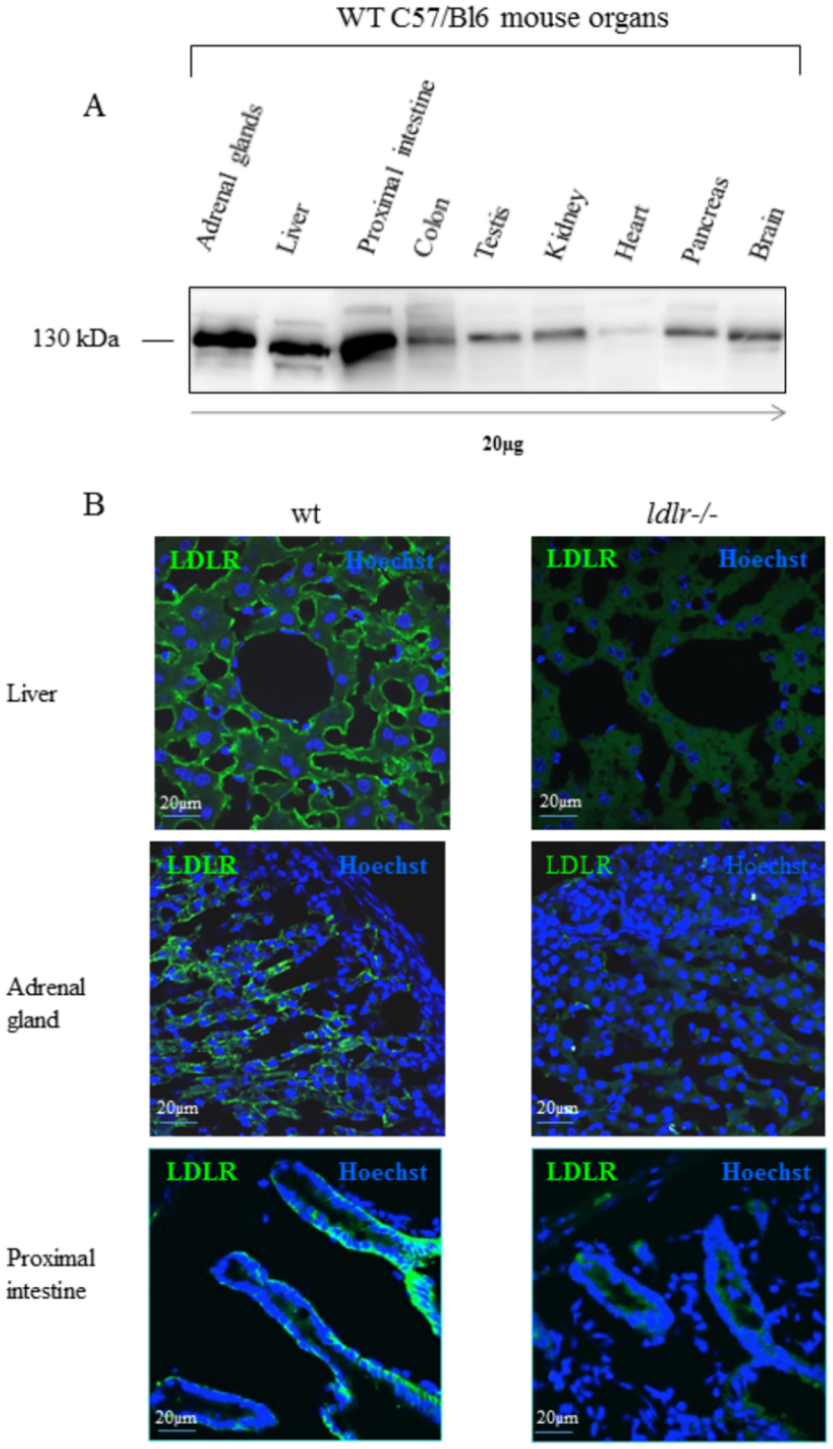
Expression of LDLR in mice organs. (A) Western Blot detection of mLDLR after migration on SDS-PAGE of 20 μg of total membrane proteins from different mouse organs. (B) Immunohistochemical detection of mLDLR (green) on cryosections (14 μm) of liver, adrenal gland and proximal intestine of WT and *ldlr-/-* mice. Cell nuclei are labeled with Hoechst#33258 (blue).

**Fig 8:**
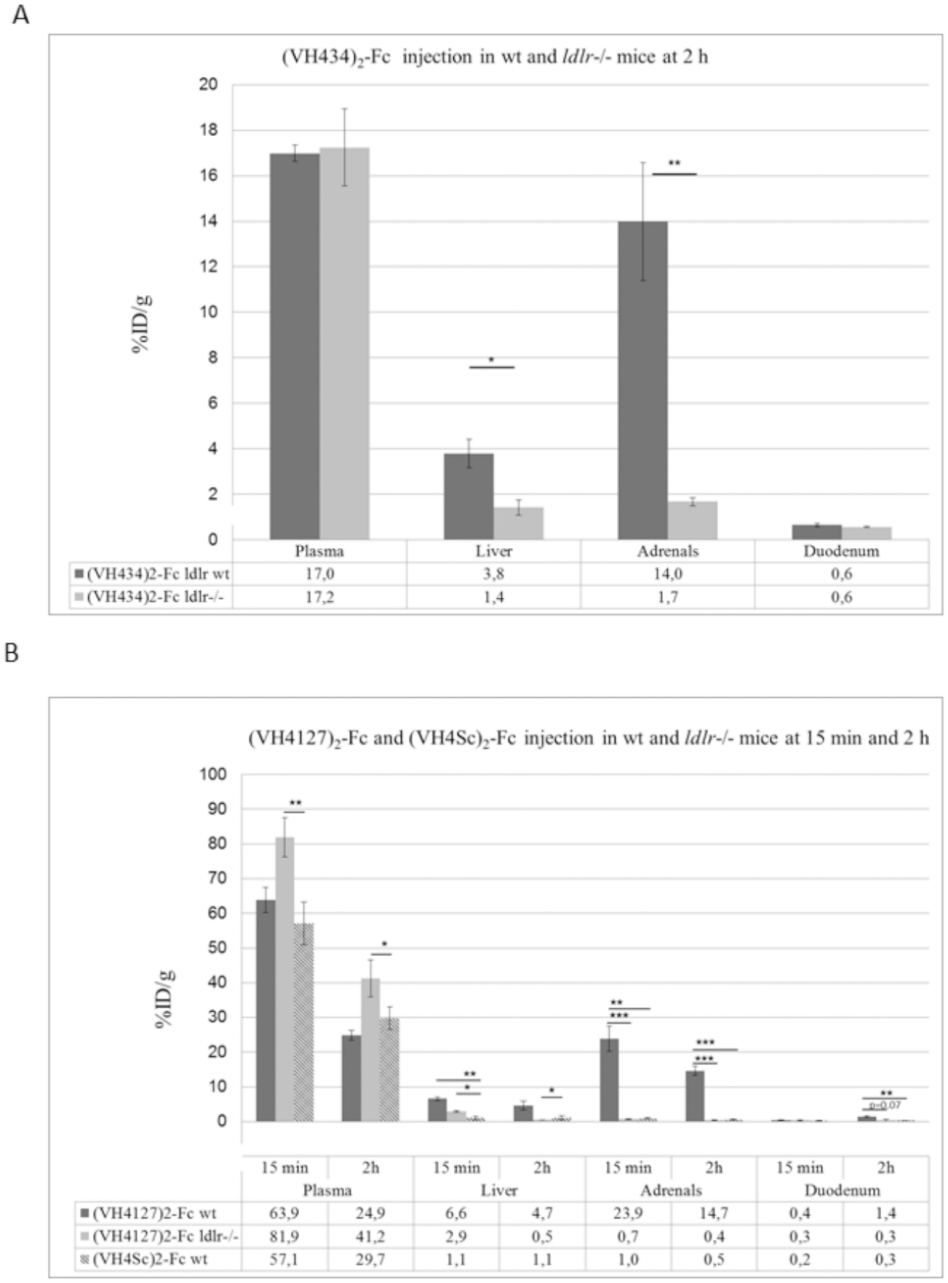
Distribution of Fc-fusion/conjugates in WT C57B/6 and *ldlr-/-* mice at 15 min and 2 hrs post-administration. (A) Percentage of injected dose per gram of tissue determined with an anti-Fc ELISA at 2 hrs following *i*.*v* injection of 0.5 mg/kg of the (VH434)_2_-Fc fusion in WT mice (dark grey bars) and *ldlr-/-* mice (light grey bars). (B) Percentage of injected dose per gram of tissue at 15 min and 2 hrs following i.v injection of 0.5 mg/kg of the (VH4127)_2_-Fc conjugate in WT mice (dark grey bars) and *ldlr-/-* mice (light grey bars), and (VH4Sc)_2_-Fc conjugate in WT mice (hatched bars). Bar graphs represent the mean of percentage of injected dose per gram of tissue ± SD (n = 3 per group and time point; *p ≤ 0.05, **p ≤ 0.01, ***p ≤ 0.001).

We evaluated the potential for the peptide-vectors to deliver large cargos into LDLR-enriched organs that were confirmed using western blot (Fig 7A) and immunohistochemistry, where LDLR distribution in liver, adrenal gland and proximal intestine was compared between WT and *ldlr-/-* mice (Fig 7B). Recombinant Fc-fusions ((VH434)_2_-Fc, (VH4Sc)_2_-Fc) were administered i.v. in WT and *ldlr-/-* mice at 0.5 mg/Kg. At different times post administration, mice were perfused with PBS, the organs of interest, namely liver, adrenal and proximal intestine, were lysed and vectorized Fc fragments were quantified using an anti-Fc ELISA. Quantification of (VH434)_2_-Fc in plasma and tissues of interest at 2 hrs post-injection in WT and *ldlr-/-* mice showed a significant 2.7-fold to 8.3 fold higher levels in the liver and adrenal glands, respectively (Fig 8A), confirming LDLR-mediated tissue uptake. Plasma levels of vectorized Fc fragments were similar in the WT and *ldlr-/-* mice.

As differences in peptide affinity and valency may modulate tissue distribution of Fc conjugates, we also analyzed the tissue distribution of (VH4127)_2_-Fc in WT and *ldlr-/-* mice at an early time point (15 min) and at 2 hrs post-injection. At both time-points, (VH4127)_2_-Fc levels were significantly lower in the plasma of WT mice compared to *ldlr-/-* mice suggesting enhanced LDLR-dependent tissue distribution in WT mice (Fig 8B). In the adrenal glands, (VH4127)_2_-Fc was ≈35-fold higher at 15 min and 2 hrs, in WT compared to *ldlr-/-* mice. In the liver, (VH4127)_2_-Fc levels were 2.3 and 9.4-fold higher in WT vs *ldlr-/-* mice at 15 min and 2 hrs respectively. Finally, in the duodenum (VH4127)_2_-Fc levels were 4.6-fold higher in WT vs *ldlr-/-* mice at 2 hrs. The tissue distribution of (VH4127)_2_-Fc *vs* (VH4Sc)_2_-Fc in WT mice was also compared at 15 min and 2 hrs post-administration. Significant fold changes in distribution were observed in the adrenal glands (≈25 fold at 15 min and 2 hrs), in the liver (6.2 fold at 15 min), and in the duodenum (4.6 fold at 2 hrs) (Fig 8B).

**Fig 9:**
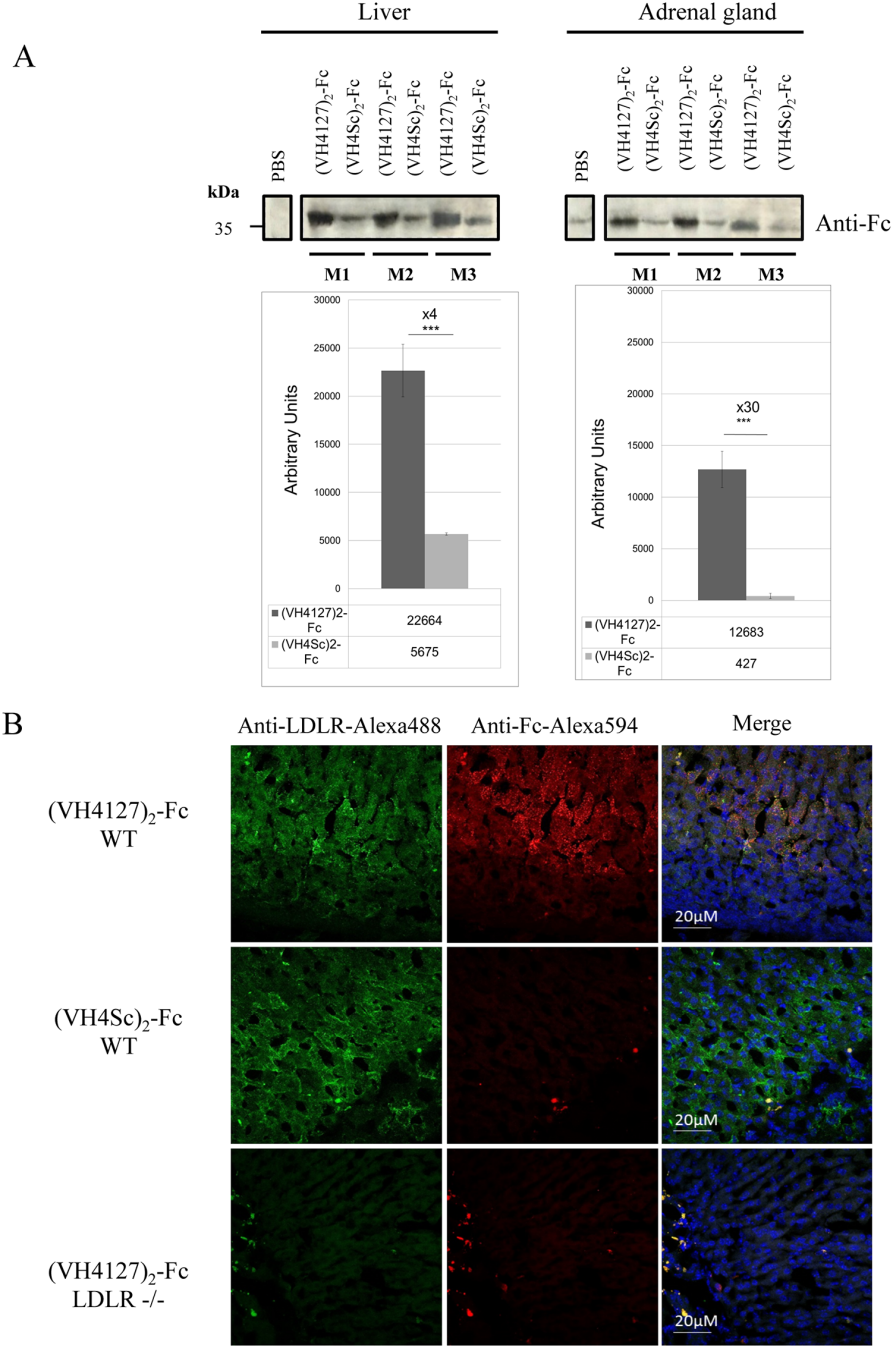
LDLR-dependent distribution of Fc-conjugates in mice organs. (A) Anti-hFc Western Blot analysis of 100 μg of liver and 35 μg of adrenal gland lysates from WT mice injected i.v. with PBS (mouse m1), with the (VH4127)_2_-Fc conjugate (mice m2, m4, m6), and with the (VH4Sc)_2_-Fc conjugate (mice m3, m5, m7). The signal detected with the PBS-injected mouse was subtracted from the signal obtained in the mice injected with the Fc-conjugate after an Image J software quantification. The mean of quantified intensity for (VH4127)_2_-Fc and (VH4Sc)_2_-Fc conjugates were plotted and compared (n = 3 per conjugate; *p ≤ 0.05, **p ≤ 0.01, ***p ≤ 0.001). (B) Immunohistochemical detection of mLDLR (green) and (VH4127)_2_-Fc and (VH4Sc)_2_-Fc conjugates (red) in WT mice, and of (VH4127)_2_-Fc in *ldlr-/-* mice (red) on 14 μm cryosections of adrenal gland at 15 min post injection of 0.5 mg/Kg conjugates. Cell nuclei were labeled with Hoechst#33258 (blue).

In order to confirm the ELISA results, tissue lysates from the liver and adrenal glands of (VH4127)_2_-Fc and (VH4Sc)_2_-Fc injected mice at 2 hrs post-administration were processed by western blot using a mouse anti-human Fc fragment antibody (Fig 9A). Intensity of the specific bands was quantified using ImageJ software. (VH4127)_2_-Fc levels were 4 and 30 times higher than (VH4Sc)_2_-Fc in the liver and adrenal glands, respectively.

Finally, tissue distribution of the (VH4127)_2_-Fc and control (VH4Sc)_2_-Fc was also assessed using immunohistochemistry on sections of PFA-fixed organs from WT and *ldlr-/-* mice following i.v. administration. Dual labeling was performed on tissue sections with antibodies directed against the human Fc and mouse LDLR. Representative photomicrographs are shown for the adrenal glands of WT and *ldlr-/-* mice at 15 min post-administration. We observed global overlap between the (VH4127)_2_-Fc signal and cells expressing LDLR in the *zona fasciculata* of WT mice. This distribution was specific since no accumulation was observed in this region for (VH4127)_2_-Fc in *ldlr-/-* mice and no Fc labeling was found in WT mice administered with the control (VH4Sc)_2_-Fc conjugate (Fig 9B).

Overall, these results confirm the LDLR-dependent tissue distribution observed with the (VH434)_2_-Fc fusion and indicate that Fc levels of accumulation can be modulated by peptide affinity and valency. In addition, the results show that all-natural amino acid peptide-vectors expressed in a fusion protein, or chemically conjugated peptides encompassing non-natural amino acids have the potential to target LDLR expressing organs *in vivo*.

## Discussion

We describe here the identification of a family of minimal sized, chemically optimized, cyclic peptide-vectors that bind to the extracellular domain of the human, mouse and rat LDLR and that do not compete with LDL, one of its main endogenous ligands. These peptide-vectors can be conjugated to a large variety of molecules, ranging from small organic molecules to siRNAs, peptides and proteins, while retaining their LDLR-binding and uptake potential *in vitro* as well as LDLR-dependent tissue targeting *in vivo*.

### Characterization of families of peptides that bind the LDLR

The LDLR has a high endocytic capacity compared to many other receptors used for organ targeting, considering that each LDLR molecule can be recycled back to the plasma membrane as much as 150 times [31]. The use of native LDL for drug targeting purposes has been demonstrated *in vitro* and *in vivo* (reviewed by [32]). Different forms of LDL particles including reconstituted LDL, synthetic nano-LDL have been proposed as versatile nanocarriers or nanoplatforms for targeted delivery of drugs, photodynamic therapy agents and diagnostics or imaging agents to LDLR-positive tissues and cancer cells [32]. However, the necessity to isolate LDL from human serum hampers its pharmaceutical application. In addition, drug delivery strategies based on apolipoproteins or full size endogenous ligands may appear difficult to implement at the industrial level and their clinical use may be further limited by saturating levels of the circulating endogenous ligands. Thus, alternative strategies were designed to reduce significantly the size and complexity of receptor targeting ligands.

Peptides are promising molecules for targeted drug delivery because of their small molecular weight, high tissue penetration, low cost of production (compared to antibodies), easy selection of candidates, fast and easy synthesis process in a homogenous form, flexibility in conjugation chemistry and poor immunogenicity [33]. The rational design of such ligands that target members of the LDLR family was implemented with the characterization of peptides that encompass consensus minimal sequences of the binding domains of endogenous protein ligands such as aprotinin (angiopep-2) [34, 35], MTf (MTfp peptide) or ApoB and ApoE binding domains (BD) [21, 36–39]. These peptides were used to address probably one of the major challenges in drug delivery and targeting, i.e. the passage across the BBB, and some of these peptides allowed peptide-drug conjugate (PDC) delivery via RMT [40–42]. One drawback of peptide-vectors designed from the receptor binding domains of endogenous protein ligands is the risk of competition with natural ligands. This may affect cell-signaling processes and induce saturation of transport capacity and receptor down-regulation over time, which might be limiting for long-term administration and delivery of an adequate and constant dosage of drugs [43, 44].

In order to identify novel peptides that do not bind the LDL binding domains, we used phage biopanning on hLDLR expressed by an engineered cell line. This strategy led to the characterization of linear and cyclic peptides that bind to different domains of the LDLR. Using LDLR expressing cell lines instead of the purified LDLR ectodomain for *in vitro* biopanning presents advantages: they retain their native states with correct protein folding, quaternary structure, and interaction with partner proteins [45]. All the cyclic peptides we identified carried a consensus tri-peptide Met-Pro-Arg (MPR) motif, flanked by 2 Cys residues and having the following general formula: (X)_n_C(X)_n_MPR(X)_n_C(X)_n_. Interestingly, some of the cyclic peptides (i.e. VH101, VH411) were identified from phage libraries constructed to encode linear peptides, arguing for the necessity of the 2 Cys residues flanking the conserved MPR motif for hLDLR binding. Phage displaying the cyclic peptides did not compete with LDL while the opposite was true for the linear peptides. The absence of competition of the cyclic peptides with LDL was further confirmed using pull down assays. These experiments showed that peptides derived and optimized from the initial VH411 peptide, such as VH445, readily bound to the EGF precursor homology domain of LDLR mini-receptors deleted for the LDL binding domain. The VH445 and VH4127 peptides derived from the VH411 peptide and chemically optimized as we detailed previously (Malcor et al., 2012; Jacquot et al., 2016), show affinities in the same range for the human and mouse LDLR. Therefore, the same molecules are amenable to both preclinical and clinical development with no need for any “drug humanization” process.

The biochemical properties of peptides can be improved by incorporating D-enantiomeric amino acids. We thus show that the affinities of the peptide-vectors we developed differ substantially depending on whether they encompass natural or non-natural amino acids. The peptides we developed show affinities up to 10 nM as monovalent peptide-vectors and can reach the sub-nanomolar range when used as multivalent conjugates, similar to that of typical therapeutic antibodies. In our study, we compared the above-mentioned ApoB, ApoE1 and ApoE2 peptides with those we describe herein (i.e. VH4127) in LDLR cell binding and endocytosis assays and show that the latter co-localized best with the LDLR and demonstrated the highest apparent affinities. Our results are in phase with the low to high μM range affinities of related ApoB and ApoE3 lipoprotein mimicking peptides [46].

We also show that affinity can be modulated by dimerization of the VH445 or VH4127 peptides. Indeed, the biological activity of peptides can be potentiated by joining monomeric peptides into di-/tri- or higher oligomeric forms, which has the potential to increase further affinity constants for target proteins as shown for the prostate-specific membrane antigen [26] and the integrin α_v_β_3_ [25]. Two of the most advanced drugs developed by screening phage-displayed libraries of random peptides are the EpoR [47] and TpoR [48] peptide agonists that target the erythropoietin and thrombopoietin receptors for the treatment of anemia and idiopathic thrombocytopenic purpura respectively. In this particular example of therapeutically active peptides, it was shown that their dimerization allowed significant improvement of their potency [47–50]. Of note, the possible benefit from multimerization of our LDLR-targeting peptides may differ depending on the pharmacology of the active drugs to which they are conjugated. Phage display also led to the identification of the HAIYPRH (T7) and THRPPMWSPVWP peptides that target the TfR [51]. A THRPPMWSPVWP-GFP fusion shows internalization into cells expressing the TfR and a TfR-lytic hybrid peptide was designed showing cytotoxic activity in 12 cancer cell lines [52].

### Conjugation versatility of peptide-vectors demonstrated *in vitro*

We demonstrated that the family of peptides we identified has high conjugation and affinity versatility. They can be conjugated to different classes of molecules ranging from small organic molecules such as fluorophores, to siRNAs, peptides or proteins, using a human IgG1-Fc fragment as a prototypic protein. In most cases, both the C and N-ter of our peptides can be used while retaining their binding properties to the LDLR, in relation to the fact that these reactive sites are in close vicinity due to the presence of a disulfide bond between the first and the last residue. Furthermore, conjugates can also encompass different types of linkers such as PEG or amino acid-based, thus enhancing the potential of the peptides for conjugation to a variety of molecules. The potential of the family of peptides we describe also resides in the fact that proteins can be functionalized either by genetic engineering with insertion of the peptide moiety in the protein sequence (example of the VH434-Fc fusion protein) or by chemical conjugation of the peptides (all natural amino-acids or not). In the latter case, different options can be used, with random conjugation to lysines or cysteines of the proteins, using bioconjugation strategies (example of VH4127-Fc “on lysine” conjugates), or enzymatic site-directed conjugation. Our family of peptides is also versatile in terms of affinity, raising the possibility to modulate not only receptor engagement in different tissues and as a consequence, levels of endocytosis, but also intracellular trafficking and cargo release in cells expressing the LDLR. For example, we show elsewhere (Varini et al., manuscript in preparation) that high affinity and/or increased avidity can lead to strong cell-surface target engagement and fast saturation of the receptor, as typically warranted in imaging approaches. On the contrary, moderate to lower affinity/avidity conjugates are more amenable to dissociate from LDLR in sorting endosomes. This in turn could provide a good prerequisite for lysosomal targeting of PDCs encompassing an organic anti-cancer drug, or a vectorized form of a lysosomal enzyme for enzyme replacement therapy in lysosomal storage diseases.

### Highly specific delivery of protein cargos to LDLR expressing tissues *in vivo*

We show that our peptide-vectors, either in fusion in the C-ter with the VH434 peptide, or chemically conjugated to the VH4127 can deliver a protein cargo such as an IgG1-Fc fragment into LDLR-enriched organs such as the adrenal gland. Comparing conjugate distribution in WT and *ldlr-/-* mice demonstrated unambiguously LDLR target engagement of the peptide-vectors with target specificity and high tissue delivery efficacy. This is in agreement with our recent results showing that the free tritium-labelled VH4127 peptide specifically accumulates in LDLR-enriched tissues in mice as early as 10 min following i.v. administration of WT compared to *ldlr-/-* mice [24]. Our results show promising potential for the peptide-vectors we developed for delivery of therapeutic molecules in LDLR expressing organs or tumors. VH445 that we initially described as “peptide 22” in an earlier study [23] was used to functionalize nanoparticles [53] and liposomes [54] loaded with fluorescent dyes, or paclitaxel and doxorubicin respectively. Functionalization of paclitaxel-loaded nanoparticles and doxorubicin-loaded liposomes increased targeting of LDLR expressing gliomas implanted in mouse brains and enhanced survival rate of mice [53], [54]. Some of our peptide-vectors (VH434 and VH4127) were shown recently to promote protein cargo (Fc fragment) transcytosis across the BBB, both *in vitro* and *in vivo*, with conjugate delivery into mouse brain (Molino et al., 2017). Our peptide-vectors could also be particularly interesting for the delivery of siRNAs or ASOs to LDLR-enriched organs as attempted previously with conjugation to cholesterol/LDL [55, 56]. Trafficking of ASGPR receptor-targeted, GalNac-vectorized, highly stable siRNAs and ASOs to endosomal or lysosomal compartments, from which these molecules can leak out to elicit their effects on gene regulation [57–59] shows promise in the preclinical and clinical settings, and the peptide-vectors we developed could open complementary avenues. Finally, many lysosomal storage diseases affect organs that express the LDLR and vectorized proteins can be advantageous in enzyme replacement therapies.

## Conclusion

Targeting cells and organs to overcome cellular barriers and promote intracellular drug delivery is a major challenge and the development of ligands that target specific cell-surface receptors involved in endocytosis is a promising strategy. The expression of LDLR by a number of tissues and lysosomal trafficking of its main ligands may open some avenues for the intracellular delivery of drugs suffering from poor membrane penetration or tissue specificity, such as many anticancer drugs, RNA-based biotherapeutics or lysosomal enzymes. Interestingly, the high LDLR expression described in some tumor cells also raises the possibility of targeting imaging agents endowed with high signal-to-noise ratio. Finally, the LDLR is expressed at the BBB and is an interesting target for transcytotic transport of drugs into the CNS [17, 60].

## Supporting information

S1 Fig Scheme describing the overall approach for identification, optimization and *in vitro/vivo* validation of LDLR targeting peptide vectors and conjugates. Step1: Hit peptides that recognize and bind to the hLDLR expressed in stable CHO cell lines are identified by screening of bacteriophage libraries presenting cyclic and linear peptides. Step2: Peptides identified in the screening process are synthetized, validated for binding to LDLR expressed in CHO cell lines stably expressing human or mouse LDLR and coated onto Biacore^™^ sensorchips, and chemically optimized. Step3: Lead peptides are conjugated to small organic molecules, siRNAs, peptides or proteins in monovalent or multivalent mode and validated for their potential to deliver their cargo *in vitro* and *in vivo*.
(PDF)

S2 Fig SPR measurement of free and conjugated peptides binding on immobilized LDLR. LDLR was captured on NiHC1000m sensor chips and increasing concentrations of analytes (50–800 nM for VH411, VH411-S-Tag, VH04sc, VH445, VH445-S-Tag and VH434; 10–160 nM for VH4127, VH4127-S-Tag, Cy5.5-PEG6-VH4127 and SiGLO CyclophilineB-VH4127; 1–16 nM for VH445 Dimer; 0.125–2 nM for VH4127 Dimer) were sequentially injected over flow cells. The black lines represent the specific binding of molecules obtained after double subtraction of the signal measured on the control flow cell (without immobilized LDLR) and a blank run. The red lines represent the fit of the data with a kinetic titration 1:1 interaction model except for VH445 and VH4127 Dimers where a bivalent analyte model was applied. The illustrated data are representative of at least 2 independent experiments.
(PDF)

S3 Fig Intracellular delivery of vector-cargo conjugates in endo-lysosomal vesicular compartments in CHO-hLDLR-EGFP cells. (A) Pulse-chase analysis of the lysosomal delivery of DiI-LDL (red). DiI-LDL 20 μg/mL was incubated on CHO-hLDLR-EGFP cells for 30 min at 4°C (pulse). At the end of a 3 hr incubation period in ligand-free medium (chase), LysoTracker^®^ DND-22 (blue) was added to the medium and incubated for an additional 5 min to stain acidic late and lysosomal compartments. Confocal images of live cells show DiI-LDL delivery to late compartments after 3 hr chase. (B) Pulse-chase analysis of the lysosomal delivery of Cy5.5-PEG6-VH4127 (blue). The Cy5.5-PEG6-VH4127 conjugate was incubated at 10 μM together with DiI-LDL 20 μg/mL (red) on CHO-hLDLR-EGFP cells for 30 min at 4°C (pulse). Following a 3 hr chase period in ligand-free medium, cells were fixed and analyzed using laser-scanning confocal microscopy. Shown is a representative image taken at the z-plane of maximal Cy5.5 intensity, demonstrating significant delivery to DiI-LDL positive compartments.
(PDF)

S1 Table Identity, sequences and mass of LDLR targeting peptides, free or conjugated/fused to different molecules. (PDF)
