# Supplementary material for: Identification and characterization of highly versatile peptide-vectors that bind non-competitively to the low-density lipoprotein receptor for in vivo targeting and delivery of small molecules and protein cargos
Source: PLoS One. 2018 Feb 27;13(2):e0191052. doi: 10.1371/journal.pone.0191052 (PMC5828360; doi:10.1371/journal.pone.0191052)
Supplement: S1 Fig — Step1: Hit peptides that recognize and bind to the hLDLR expressed in stable CHO cell lines are identified by screening of bacteriophage libraries presenting cyclic and linear peptides. Step2: Peptides identified in the screening process are synthetized, validated for binding to LDLR expressed in CHO cell lines stably expressing human or mouse LDLR and coated onto Biacore™ sensorchips, and chemically optimized. Step3: Lead peptides are conjugated to small organic molecules, siRNAs, peptides or proteins in monovalent or multivalent mode and validated for their potential to deliver their cargo in vitro and in vivo. (PDF) [file pone.0191052.s001.pdf]

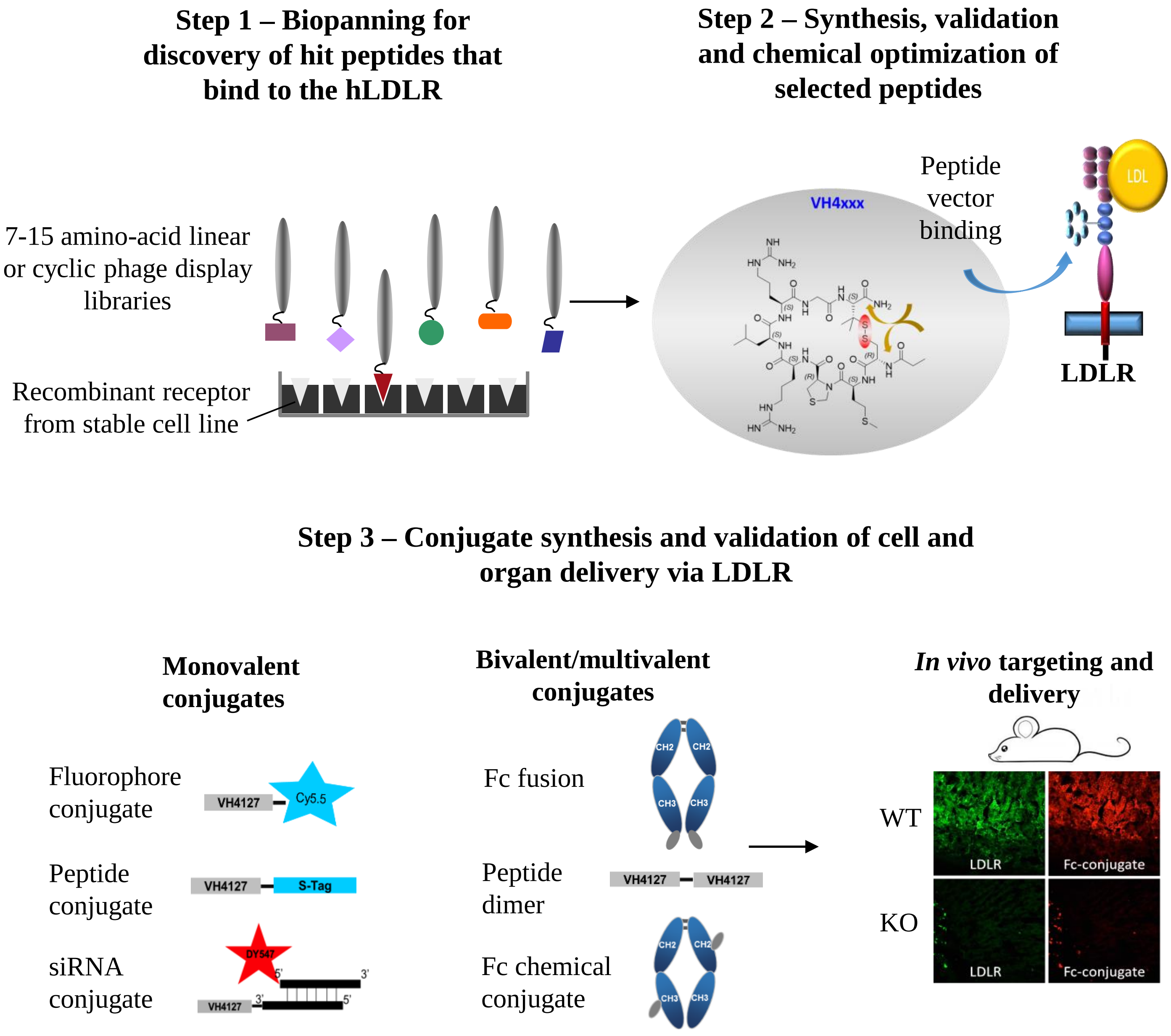

S1 Fig: Scheme describing the overall approach for identification, optimization and *in vitro/vivo* validation of LDLR targeting peptide vectors and conjugates. Step1: Hit peptides that recognize and bind to the hLDLR expressed in stable CHO cell lines are identified by screening of bacteriophage libraries presenting cyclic and linear peptides**.** Step2: Peptides identified in the screening process are synthetized, validated for binding to LDLR expressed in CHO cell lines stably expressing human or mouse LDLR and coated onto Biacore^{TM} sensorchips, and chemically optimized. Step3: Lead peptides are conjugated to small organic molecules, siRNAs, peptides or proteins in monovalent or multivalent mode and validated for their potential to deliver their cargo *in vitro* and *in vivo*.
