# Supplementary material for: Identification and characterization of highly versatile peptide-vectors that bind non-competitively to the low-density lipoprotein receptor for in vivo targeting and delivery of small molecules and protein cargos
Source: PLoS One. 2018 Feb 27;13(2):e0191052. doi: 10.1371/journal.pone.0191052 (PMC5828360; doi:10.1371/journal.pone.0191052)
Supplement: S2 Fig — LDLR was captured on NiHC1000m sensor chips and increasing concentrations of analytes (50–800 nM for VH411, VH411-S-Tag, VH04sc, VH445, VH445-S-Tag and VH434; 10–160 nM for VH4127, VH4127-S-Tag, Cy5.5-PEG6-VH4127 and SiGLO CyclophilineB-VH4127; 1–16 nM for VH445 Dimer; 0.125–2 nM for VH4127 Dimer) were sequentially injected over flow cells. The black lines represent the specific binding of molecules obtained after double subtraction of the signal measured on the control flow cell (without immobilized LDLR) and a blank run. The red lines represent the fit of the data with a kinetic titration 1:1 interaction model except for VH445 and VH4127 Dimers where a bivalent analyte model was applied. The illustrated data are representative of at least 2 independent experiments. (PDF) [file pone.0191052.s002.pdf]

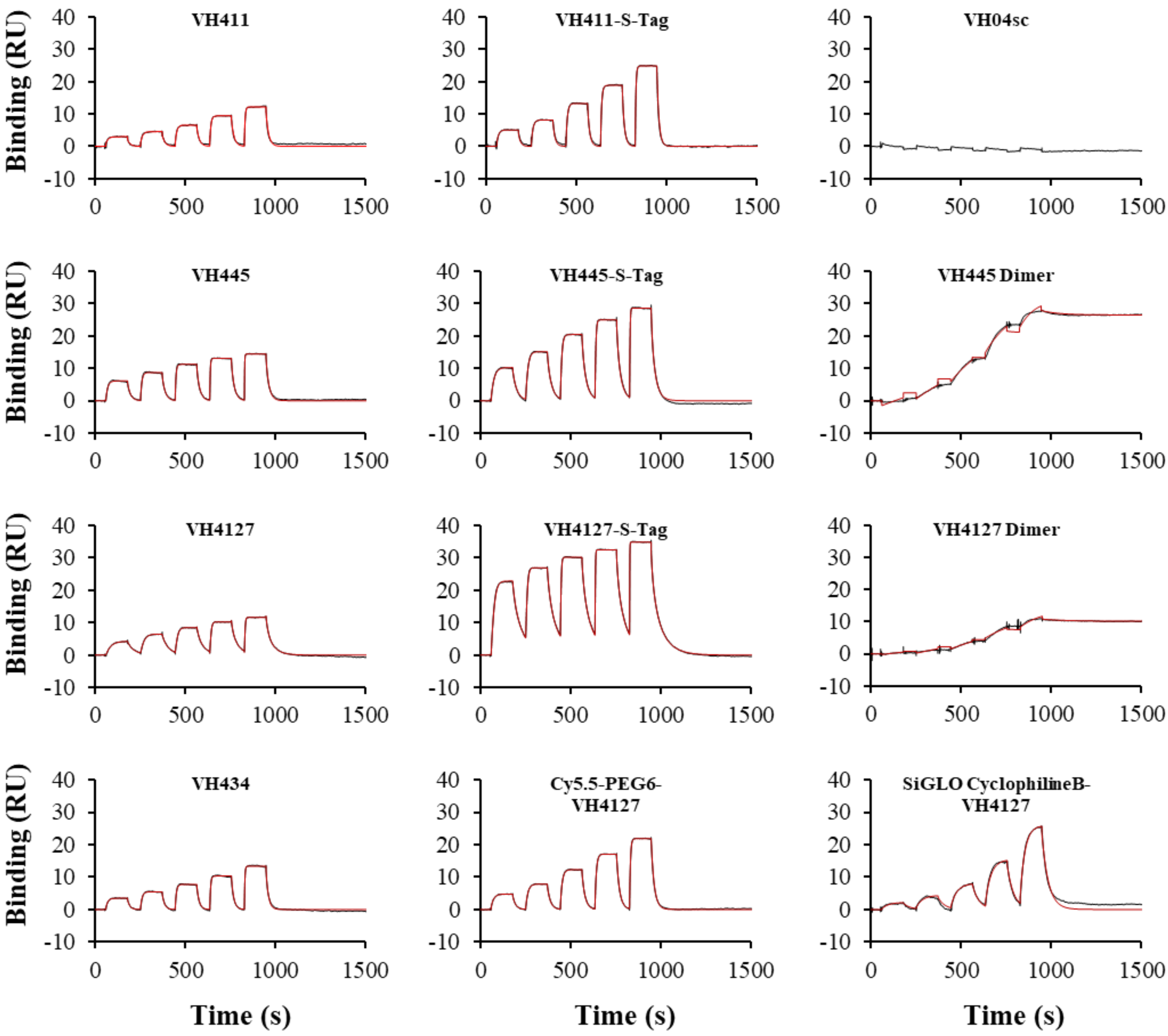

S2 Fig: SPR measurement of free and conjugated peptides binding on immobilized LDL-R. LDL-R was captured on NiHC1000m sensor chip and increasing concentrations of analytes (50-800 nM for VH411, VH411-S-Tag, VH04sc, VH445, VH445-S-Tag and VH434; 10-160 nM for VH4127, VH4127-S-Tag, Cy5.5-PEG6-VH4127 and SiGLO CyclophilineB-VH4127; 1-16 nM for VH445 Dimer; 0.125-2 nM for VH4127 Dimer) were sequentially injected over flow cells. The black lines represent the specific binding of molecules obtained after double subtraction of the signal measured on the control flow cell (without immobilized LDL-R) and a blank run. The red lines represent the fit of the data with a kinetic titration 1:1 interaction model excepted for VH445 and VH4127 Dimers where a bivalent analyte model was applied. The illustrated data are representative of at least 2 independent experiments.
