# Supplementary material for: Identification and characterization of highly versatile peptide-vectors that bind non-competitively to the low-density lipoprotein receptor for in vivo targeting and delivery of small molecules and protein cargos
Source: PLoS One. 2018 Feb 27;13(2):e0191052. doi: 10.1371/journal.pone.0191052 (PMC5828360; doi:10.1371/journal.pone.0191052)
Supplement: S3 Fig — (A) Pulse-chase analysis of the lysosomal delivery of DiI-LDL (red). DiI-LDL 20 μg/mL was incubated on CHO-hLDLR-EGFP cells for 30 min at 4°C (pulse). At the end of a 3 hr incubation period in ligand-free medium (chase), LysoTracker® DND-22 (blue) was added to the medium and incubated for an additional 5 min to stain acidic late and lysosomal compartments. Confocal images of live cells show DiI-LDL delivery to late compartments after 3 hr chase. (B) Pulse-chase analysis of the lysosomal delivery of Cy5.5-PEG6-VH4127 (blue). The Cy5.5-PEG6-VH4127 conjugate was incubated at 10 μM together with DiI-LDL 20 μg/mL (red) on CHO-hLDLR-EGFP cells for 30 min at 4°C (pulse). Following a 3 hr chase period in ligand-free medium, cells were fixed and analyzed using laser-scanning confocal microscopy. Shown is a representative image taken at the z-plane of maximal Cy5.5 intensity, demonstrating significant delivery to DiI-LDL positive compartments. (PDF) [file pone.0191052.s003.pdf]

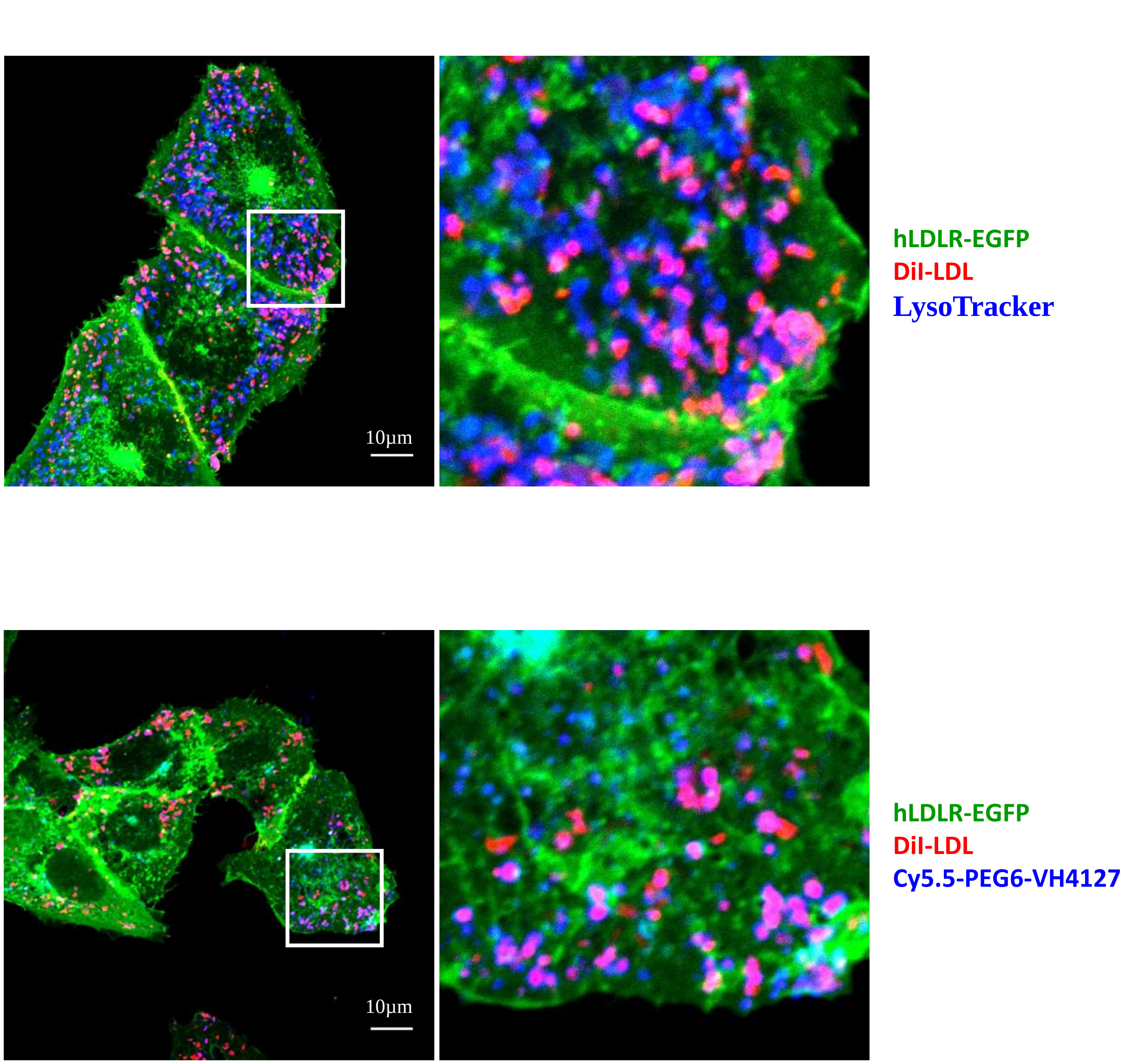

S3 Fig: Intracellular delivery of vector-cargo conjugates in endo-lysosomal vesicular compartments in CHO-hLDLR-EGFP cells. (A) Pulse-chase analysis of the lysosomal delivery of DiI-LDL (red). DiI-LDL 20 µg/mL was incubated on CHO-hLDLR-EGFP cells for 30 min at 4°C (pulse). At the end of a 3 hr incubation period in ligand-free medium (chase), LysoTracker® DND-22 (blue) was added to the medium and incubated for an additional 5 min to stain acidic late and lysosomal compartments. Confocal images of live cells show DiI-LDL delivery to late compartments after 3 hr chase. (B) Pulse-chase analysis of the lysosomal delivery of Cy5.5-PEG6-VH4127 (blue). The Cy5.5-PEG6-VH4127 conjugate was incubated at 10 µM together with DiI-LDL 20 µg/mL (red) on CHO-hLDLR-EGFP cells for 30 min at 4°C (pulse). Following a 3 hr chase period in ligand-free medium, cells were fixed and analyzed using laser-scanning confocal microscopy. Shown is a representative image taken at the z-plane of maximal Cy5.5 intensity, demonstrating significant delivery to DiI-LDL positive compartments.
