# Supplementary material for: Identification and characterization of highly versatile peptide-vectors that bind non-competitively to the low-density lipoprotein receptor for in vivo targeting and delivery of small molecules and protein cargos
Source: PLoS One. 2018 Feb 27;13(2):e0191052. doi: 10.1371/journal.pone.0191052 (PMC5828360; doi:10.1371/journal.pone.0191052)
Supplement: S1 Table — (PDF) [file pone.0191052.s004.pdf]

**S1 Table: Sequences, purity and identity of LDLR targeting peptides, free or conjugated/fused to different molecules.**

| Peptide | Sequence [a], Formula | (m/z) expected | (m/z) found |
|---|---|---|---|
| VH411 | Ac-DSGLCMPRLRGCDPR-$NH_2$ $C_{68}H_{115}N_{25}O_{21}S_3$ | [M+2H]2+ 857.8 | [M+2H]2+ 858.5[b] |
| VH434 | Pr-CMPRLRGC-$NH_2$ $C_{39}H_{69}N_{15}O_9S_3$ | [M+H]+ 988.46 | [M+H]+ 988.61[b] |
| VH445 | Pr-cMPRLRGC-$NH_2$ $C_{39}H_{69}N_{15}O_9S_3$ | [M+H]+ 988.46 | [M+H]+ 988.45[c] |
| VH4127 | Pr-cMThzRLRG"Pen"-$NH_2$ $C_{40}H_{71}N_{15}O_9S_4$ | [M+H]+ 1034.44 | [M+H]+ 1034.11[b] |
| VH411-S-Tag | Ac-DSGLCMPRLRGCDPR-GGG-KETAAAKFERQHMDS-$NH_2$ $C_{147}H_{238}N_{50}O_{49}S_4$ | [M+3H]3+ 1205.9 | [M+3H]3+ 1206.6[b] |
| VH445-S-Tag | Pr-cMPRLRGC-GGG-KETAAAKFERQHMDS-$NH_2$ $C_{118}H_{193}N_{41}O_{36}S_4$ | [M+2H]2+ 1445.17 | [M+2H]2+ 1446,17[b] |
| VH4127-S-Tag | Pr-cM"Thz"RLRG"Pen"-GGG-KETAAAKFERQHMDS-$NH_2$ $C_{119}H_{195}N_{41}O_{36}S_5$ | [M+H]+ 2935,33 | [M+H]+ 2935.32[c] |
| Cy5.5-PEG6-VH4127 | Cy5.5-PEG6-cM"Thz"RLRG"Pen"-$NH_2$ $C_{92}H_{137}N_{18}O_{28}S_{8}^{+}$ | [M+2H]2+ 1471.8 | [M+2H]2+ 1471.5[d] |
| VH445 Dimer | (Pr-cMPRLRGC-G-NH-$CH_2$-$CH_2)_2$-N-$(CH_2)_2$-NH2 $C_{88}H_{156}N_{34}O_{20}S_6$ | [M+H]+ 2202.9 | [M+H]+ 2203.98[c] |
| VH4127 Dimer | (Pr-cM"Thz"RLRG"Pen"-G-NH-$CH_2$-$CH_2)_2$-N-$(CH_2)_2$-$NH_2$ $C_{90}H_{160}N_{34}O_{20}S_8$ | [M+H]+ 2294,03 | [M+H]+ 2294.20[c] |

(a) One-letter amino acid code; otherwise specified (non-natural amino acids), lower case letters indicate (D)-configuration. “Ac-” and “Pr-” mean N-terminal acetylation and propionylation, respectively. (b) ESI mass spectrometry performed with a LCQ Fleet ThermoFisher used in positive mode (c) MALDI-TOF-TOF Ultraflex II Bruker used in positive reflectron mode, matrix: alpha-cyano-4-hydroxycinnamic acid (CHCA). (d) ESI mass spectrometry performed with a Bruker UltrafleXtreme used in positive mode.

| Peptide-Fc conjugate | Sequence [a] | « nude » Fc | Δ (m/z) expected | (m/z) found[e] | Δ (m/z) found | PAR found |
|---|---|---|---|---|---|---|
| $(VH434)_2$-Fc | hIgG1Fc-GGG-$(CMPRLRGC)_2$ | 55639.0[b] | 2204.8 | 57798.5 | 2159.5 | 2.0 |
| $(VH04sc)_2$-Fc | hIgG1Fc-SMCC-( Pr-cRPLGRMC-G-$(CH_2)_2$-S$)_2$ | 53180.7[c] | 2646.8 | 55869.3 | 2688.6 | 2.0 |
| $(VH4127)_2$-Fc | hIgG1Fc-SMCC-(Pr-cM"Thz"RLRG"Pen"-G-$(CH_2)_2$-S$)_2$ | 53180.7[c] | 2739.1 | 55730.1 | 2549.3 | 1.9 |

(a) One-letter amino acid code; otherwise specified (non-natural amino acids), lower case letters indicate (D)-configuration. “Ac-” and “Pr-” mean N-terminal acetylation and propionylation, respectively. (b) Fc fragment from in-house production (c) Fc fragment from commercial source (e) MALDI-TOF-TOF Bruker UltrafleXtreme used in positive reflectron mode.
